# SOX9+/PTF1A+ Cells Define the Tip Progenitor Cells of the Human Fetal Pancreas of the Second Trimester

**DOI:** 10.1002/sctm.19-0231

**Published:** 2019-10-21

**Authors:** Valentina Villani, Matthew E. Thornton, Heather N. Zook, Christiana J. Crook, Brendan H. Grubbs, Giuseppe Orlando, Roger De Filippo, Hsun Teresa Ku, Laura Perin

**Affiliations:** ^1^ GOFARR Laboratory for Organ Regenerative Research and Cell Therapeutics, Division of Urology Saban Research Institute, Children's Hospital Los Angeles Los Angeles California USA; ^2^ Maternal‐Fetal Medicine Division, Department of Obstetrics and Gynecology, Keck School of Medicine University of Southern California Los Angeles California USA; ^3^ Department of Translational Research and Cellular Therapeutics Diabetes and Metabolism Research Institute of City of Hope Duarte California USA; ^4^ Irell & Manella Graduate School of Biological Sciences Beckman Research Institute of City of Hope Duarte California USA; ^5^ Department of Surgery Wake Forest School of Medicine Winston‐Salem North Carolina USA; ^6^ Department of Urology, Keck School of Medicine University of Southern California Los Angeles California USA

**Keywords:** Pancreas, Development, Human, Fetal, Multipotent pancreatic progenitors

## Abstract

Significant progress has been made in recent years in characterizing human multipotent progenitor cells (hMPCs) of the early pancreas; however, the identity and persistence of these cells during the second trimester, after the initiation of branching morphogenesis, remain elusive. Additionally, studies on hMPCs have been hindered by few isolation methods that allow for the recovery of live cells. Here, we investigated the tip progenitor domain in the branched epithelium of human fetal pancreas between 13.5 and 17.5 gestational weeks by immunohistological staining. We also used a novel RNA‐based technology to isolate live cells followed by gene expression analyses. We identified cells co‐expressing SOX9 and PTF1A, two transcription factors known to be important for pancreatic MPCs, within the tips of the epithelium and observed a decrease in their proportions over time. Pancreatic *SOX9*+/*PTF1A*+ cells were enriched for MPC markers, including *MYC* and *GATA6*. These cells were proliferative and appeared active in branching morphogenesis and matrix remodeling, as evidenced by gene set enrichment analysis. We identified a hub of genes pertaining to the expanding tip progenitor niche, such as *FOXF1*, *GLI3*, *TBX3*, *FGFR1*, *TGFBR2*, *ITGAV*, *ITGA2*, and *ITGB3*. YAP1 of the Hippo pathway emerged as a highly enriched component within the *SOX9*+/*PTF1A*+ cells. Single‐cell RNA‐sequencing further corroborated the findings by identifying a cluster of *SOX9*+/*PTF1A*+ cells with multipotent characteristics. Based on these results, we propose that the *SOX9*+/*PTF1A*+ cells in the human pancreas are uncommitted MPC‐like cells that reside at the tips of the expanding pancreatic epithelium, directing self‐renewal and inducing pancreatic organogenesis. stem cells translational medicine *2019;8:1249&1264*

## Abstract 2

Schematic representation of SOX9+/PTF1A+ cell isolation and characterization.

## Graphical abstract

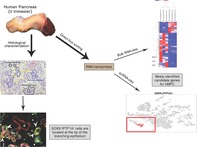

**Significance Statement**

With the use of RNA‐labeling probes, the authors report for the first time the direct isolation of live pancreatic progenitors co‐expressing SOX9 and PTF1A from human pancreas of the second trimester. Pancreatic multipotent progenitor state was confirmed by gene profiling by bulk RNA‐seq and single cell RNA‐seq. This first “snapshot” of the transcriptional network of human pancreatic progenitors opens new avenues in understanding human pancreas development, pancreatic specification and supports the ultimate goal of understanding possible mechanisms for pancreas regeneration.

## Introduction

The developing mammalian pancreas has a highly organized structure, in which subpopulations of progenitor cells and lineage‐committed cells can be identified based on their molecular profile and location within the pancreatic epithelium, following the initiation of branching morphogenesis. Pancreatic multipotent progenitor cells (MPCs) in the early mouse embryo are known to express several key transcription factors such as Pdx1, Sox9, and Ptf1a, which are indispensable for MPC establishment, maintenance, and proliferation 1, 2, 3, 4. Pdx1, a master regulator of the pancreatic program, is responsible for initiation of pancreatogenesis and is strongly expressed by all MPCs. Sox9 is crucial to maintaining the MPC niche and its role was identified through the observation of pancreatic hypoplasia in *Sox9* null mice 4 and lineage‐tracing studies 5. When branching morphogenesis initiates around e12.5 in the mouse 6, MPCs also express Ptf1a and other transcription factors and are localized at the tips of the branching epithelium. These cells remain multipotent with the potentials to give rise to the three major pancreatic lineages, that is, endocrine, acinar, and ductal cells, until they become restricted to the acinar lineage 7. Thus, Pdx1, Sox9, and Ptf1a constitute core transcription factors of murine MPCs that are necessary to determine pancreatic development.

In humans, natural genetic mutations of PDX1, SOX9, or PTF1A have been found that lead to pancreatic agenesis 8, 9, 10, 11 or neonatal diabetes and impaired pancreatic development 12, 13, 14, 15, 16, similarly to what has been observed in mouse models 17, 18, 19, 20, 21. Expression of PDX1, SOX9, and PTF1A was confirmed in the human embryonic pancreas 22, 23. However, prior studies have mostly focused on the expression of PDX1 and SOX9, but not PTF1A, in human (h) MPCs. Additionally, most studies examined hMPCs during the first trimester, at 9 weeks (W) of gestational age (GA) or earlier. Less is known about whether hMPCs persist in the second trimester, well after the first appearance of endocrine cells at 8WGA 24, 25.

Importantly, studies on hMPCs have been hindered by scarce accessibility to fetal tissues and few isolation methods that allow for recovery of live cells. To isolate MPCs, most laboratories rely on the use of fluorescent reporter proteins in transgenic animal models or cell‐surface markers obtained by screening of in vitro‐derived hMPC‐like cells 26, 27, 28. We recently reported the successful isolation of live renal progenitor cells from human fetal kidney using RNA‐based fluorescent probes (Smartflare technology) combined with fluorescence‐activated cell sorting (FACS) 29. In this study, we investigated the utility of the Smartflare technology to isolate and profile *SOX9*+/*PTF1A*+ cells in the human fetal pancreas (hFP) during the second trimester (from 13.5 to 17.5WGA).

We report here the identification of a pool of cells residing at the tips of the branched epithelium co‐expressing SOX9 and PTF1A. Genome‐wide gene expression studies revealed that these cells express markers of uncommitted MPCs, are proliferative, and contribute to branching morphogenesis. Single‐cell RNA profiling of a 15.4WGA pancreas further confirm an MPC‐like signature of the SOX9+/PTF1A+ cells.

To the best of our knowledge, this is the first report that identifies the location and the genetic profile of hMPCs characterized by the expression of SOX9 and PTF1A in the second trimester hFP. Our work contributes to the understanding of human pancreatic development and pancreatic cell specification, and has potential implications in advancing in vitro methods to generate hMPCs on large scale and derive functional insulin‐secreting cells suitable for clinical application.

## Materials and Methods

### hFP Tissue Collection and Digestion

After informed consent, deidentified hFP samples were collected under the Institutional Review Board's approval of the University of Southern California and the Children's Hospital Los Angeles. The only information collected was gestational age and whether there were any known genetic or structural abnormalities. A total of *n* = 22 samples between 13.5 and 17.5WGA were used. Specifically, for histological analysis, *n* = 5 samples were used; for flow cytometry analysis and probe testing, *n* = 8 samples were used. For FACS sorting and bulk RNA‐seq, *n* = 8 samples were used. Single‐cell (sc)RNA‐seq was performed on *n* = 1 hFP at 15.4WGA. In addition, human fetal lung cells (approved under the same IRB protocol) were collected and used as control for FGFR2 antibody specificity.

To obtain single‐cell suspensions, each hFP was digested in 1 mg/ml collagenase P (Roche, Basel, Switzerland) dissolved in Medium 199 (Life Technologies, Carlsbad, CA, USA) for 20 minutes at 37°C, then washed twice in PBS containing DNAse I (Thermo Fisher Scientific, Waltham, MA, USA), and incubated with trypsin for 5 minutes and either processed for flow cytometry analysis or plated in Medium 199 supplemented with 10% fetal bovine serum (ES cell grade, Life Technologies, Carlsbad, CA, USA), 1% Penicillin–Streptomycin (Life Technologies, Carlsbad, CA, USA), and 0.2% Primocin (InvivoGen, San Diego, CA, USA) for RNA probe selection. The same digestion protocol was used to obtain a single‐cell suspension for scRNA‐seq, except cells were washed in PBS1X without DNAse I.

### Histology and Immunohistochemistry

hFP tissues were fixed, dehydrated, paraffin‐embedded, and H&E stained as previously 30. Images were acquired with a Leica DM1000 microscope. For immunohistochemistry, heat‐mediated antigen retrieval was performed in a citrate‐based solution (Vector Laboratories, Burlingame, CA, USA), followed by blocking in PBS containing 2% BSA for 30 minutes at room temperature (RT). The primary antibodies and dilutions used are listed in Table 1.

**Table 1:** List of antibodies used for immunohistochemistry and flow cytometry

Target	Vendor	Cat. N.	Application	Dilution	Incubation
E‐cadherin	Abcam	ab45990	IHC	1/200	1 hour, RT
PTF1A	Donated by Dr. Mastracci	n/a	IHC	1/500	4°C, ON
SOX9	Abnova	H00006662‐M02	IHC	1/100	2 hours, RT
PDX1	Abcam	ab47383	IHC	1/500	1 hour, RT
FGFR2	Abcam	ab10648	IHC	1/500	1 hour, RT
PCNA	Abcam	ab2426	IHC	1/1,000	1 hour, RT
Glucagon	Abcam	ab8055	IHC	1/100	1 hour, RT
Amylase	Donated by Dr. Mastracci	n/a	IHC	1/500	4°C, ON
CPA1	Novus Biologicals	NBP1‐47704	IHC	1/500	1 hour, RT
SOX9	Abcam	ab196450	FC	1/50 × 10^7^ cells	30 minutes, ice
PTF1a	BD Pharmingen	564750	FC	1/20 × 10^7^ cells	30 minutes, ice
FGFR2	Novus Biologicals	FAB6843G	FC	1/10 × 10^6^ cells	1 hour, ice
Mouse IgG1	R&D systems	MAB002	FC	1/25 × 10^6^ cells	1 hour, ice

Antibodies were incubated for 1 hour at RT or overnight at 4°C, following the manufacturer's instructions. Host‐specific Alexa Fluor‐conjugated secondary antibodies (anti‐mouse Alexa Fluor 555, anti‐rabbit Alexa Fluor 488, and anti‐goat Alexa Fluor 647; Thermo Fisher Scientific, Waltham, MA, USA) were applied for 30 minutes at RT. Sections were mounted with Vectashield medium containing DAPI (Vector Laboratories, Burlingame, CA, USA) to stain nuclei. Antibodies against PDX1 and PTF1A were both raised in goat. To avoid cross‐reaction in the triple staining of SOX9/PDX1/PTF1A, antibodies against PDX1 were directly conjugated with Zenon Alexa Fluor 647 using a Goat IgG Labeling Kit (Thermo Fisher Scientific, Waltham, MA, USA) (PDX1‐AF647). To perform immunocytochemistry, following ON incubation with the RNA probe, cells were fixed in 4% paraformaldehyde (Santa Cruz Biotechnology, Dallas, TX, USA) for 10 minutes and permeabilized with PBS containing 0.1% Triton X‐100 (Alfa Aesar, Haverhill, MA, USA). Staining was then performed using a SOX9 antibody. Images were acquired with a Leica fluorescent microscope (Model DFC360 FX).

### Smartflare Technology, FACS Sorting, and Flow Cytometry

Dissociated hFP cells were incubated ON with both SOX9‐cyanine 5 (Cy5) and PTF1A‐cyanine 3 (Cy3) Smartflare RNA probes (SF1011‐ and SFC‐1; EMD Millipore, Billerica, MA, USA http://www.millipore.com). Probes were diluted 1:20 in PBS and incubated ON. A Scramble probe (negative control, SF‐102; EMD Millipore, Billerica, MA, USA) and uptake probes (positive control; Cy3 SF‐114, Cy5 SF‐137; EMD Millipore) were used. A probe specific for kidney progenitor cells (CITED1, SFC‐319; EMD Millipore, Billerica, MA, USA) was used to further validate the specificity of our probes.

Smartflare‐treated cells were also prepared for sorting by blocking with a solution of human IgG (100 μl/up to 10^6^ cells) for 10 minutes, followed by incubation with anti‐FGFR2 antibody (Novus Biologicals, Centennial, CO, USA, 2 μg/10^6^ cells) for 1 hour. Cells were sorted using a FACSAria (Becton Dickinson [BD], Franklin Lakes, NJ, USA). Single fluorochrome controls and Smartflare uptake probes were used to set compensation. Upon sorting, cells were immediately pelleted and frozen for subsequent RNA extraction. A mouse IgG1 isotype control (2 μg/10^6^ cells, R&D systems, Minneapolis, MN, USA) was used to validate the specificity of the FGFR2 antibody. Unstained and single‐positive controls were used to perform area scaling, excluding autofluorescence, and, when needed, fluorochrome compensation.

For flow cytometry analysis, cells were fixed and permeabilized with the eBioscience Foxp3/Transcription Factor Staining Buffer Set (Thermo Fisher Scientific, Waltham, MA, USA). Cells were then blocked in human IgG solution for 10 minutes and incubated with antibodies against SOX9 and PTF1A for 30 minutes on ice. Analysis was performed on a FACSCanto (BD) using the FACSDiva software. The gating strategy was as follows: cells were first gated based on forward and side scatter (FSC/SSC) to exclude dead cells, then gated for FSC‐Width (FSC‐W) by FSC‐Height (FSC‐H) and SSC‐Width (SSC‐W) by SSC‐Height (SSC‐H) to exclude potential doublets. Final sorting gates were established based on the unstained control for each sample (Fig. S1).

### Bulk RNA Sequencing

RNA extraction and RNA‐seq were performed by Quickbiology (Quickbiology Inc., Pasadena, CA, USA). RNA integrity was checked with an Agilent Bioanalyzer 2100. Libraries were prepared according to the QIAseq FX Single Cell RNA Library Kit (Qiagen, Hilden, Germany) with 200–300 bp insert size using 25 ng total RNA as input. Final library quality and quantity was analyzed with an Agilent Bioanalyzer 2100 and a Life Technologies Qubit3.0 Fluorometer. Paired‐end tags of 150 bp in length were sequenced and read on an Illumina HiSeq 4000 (Illumina Inc., San Diego, CA, USA).

Data processing was performed using the high‐performance computing cluster at the University of Southern California (https://hpcc.usc.edu/). Roughly, 50 million 150 bp paired‐end sequences were aligned to the version 29 Gencode genome based on Genome Reference Consortium Human build number 38 using the STAR aligner with “GeneCounts” output 31. The software Morpheus (https://software.broadinstitute.org/morpheus) was used to generate heatmaps of specific enrichment gene sets and perform hierarchical clustering. Stranded values of Reads Per Kilobase of transcript per Million mapped reads (RPKM) were used to produce the heatmaps. Differential gene expression was determined using the R/Bioconductor software “edgeR” (20). Genes are considered differentially expressed at false discovery rate (FDR)‐adjusted *p* value of <.05 and log2 fold change (log2FC) > 1.5 or less than −1.5.

Gene set enrichment analysis (GSEA) was performed on differentially expressed genes between SOX9+/PTF1A+ and SOX9−/PTF1A− cells (*n* = 3 donors). A total of 2,233 genes were entered into the Broad Institute javaGSEA Desktop application (http://software.broadinstitute.org/gsea/downloads.jsp) as an expression data set. The data set was filtered from an initial 21,876 differentially expressed molecules based on: protein coding genes, *p* < .05, FDR *p* < .05, and log2FC either <−1.5 or >1.5. The number of permutations was set to the default (1,000), and the permutation type was set to gene set due to having *n* = 3 samples per phenotype. The C5 gene ontology (GO) gene set (C5.all.v6.2.symbols.gmt) was obtained from the Molecular Signatures Database v6.2 to analyze the 2,233 genes. The gene set size filter was set to the default (min = 15, max = 500), resulting in 1,440 gene sets used in this analysis (run *n* = 2). All other fields remained at default settings. Leading Edge Analysis was run on 6 enriched gene sets of interest within the top 45 SOX9+/PTF1A+‐enriched gene sets (*p* < .05, FDR *p* < .05, normalized enrichment score [NES] > 2.50). The NES is calculated by the software (http://software.broadinstitute.org/gsea/downloads.jsp).

### Single Cell RNA Sequencing

After single‐cell suspension of an hFP (15.4WGA), removal of red blood cells was performed using the red blood cell lysis solution (Miltenyi Biotec, Bergisch Gladbach, Germany) following manufacturer's recommendations. Approximately 9,000 cells were captured on a 10x Chromium device using a 10X V3 Single Cell 3′ Solution kit (10xGenomics, Chromium Single Cell 3’ Regent kit V3 Chemistry, Cat. PN‐1000092). All protocols were performed following the manufacturer's instructions. Final sequencing libraries were analyzed on a High Sensitivity DNA Chip (Agilent, Santa Clara, CA, USA, Cat 5067‐4626) to determine the library size; final library concentrations were determined using a Qubit high Sensitivity DNA assay Kit (Thermo Fisher Scientific, Waltham, MA, USA, Cat. Q32854). Libraries were sequenced with the paired end setting of 101–101 with 8 cycles of index read on an Illumina NovaSeq 6000 platform. Approximately, 0.1 million reads per cells were sequenced. 10X Genomics Loupe cell browser was used to visualize and analyze the final data.

### Ingenuity Pathway Analysis (IPA)

IPA (Qiagen) was used to evaluate potential biologically significant processes between SOX9+/PTF1A+ cells and SOX9−/PTF1A− cells (*n* = 3 donors). Log2FC values, obtained from RPKM generated through bulk RNA‐seq and scRNA‐seq, were uploaded to the IPA platform and overlaid with the global gene network in the Ingenuity Knowledge Base.

## Results

### In Situ Identification of SOX9+/PTF1A+ Cells in the Second Trimester

**Figure 1:**
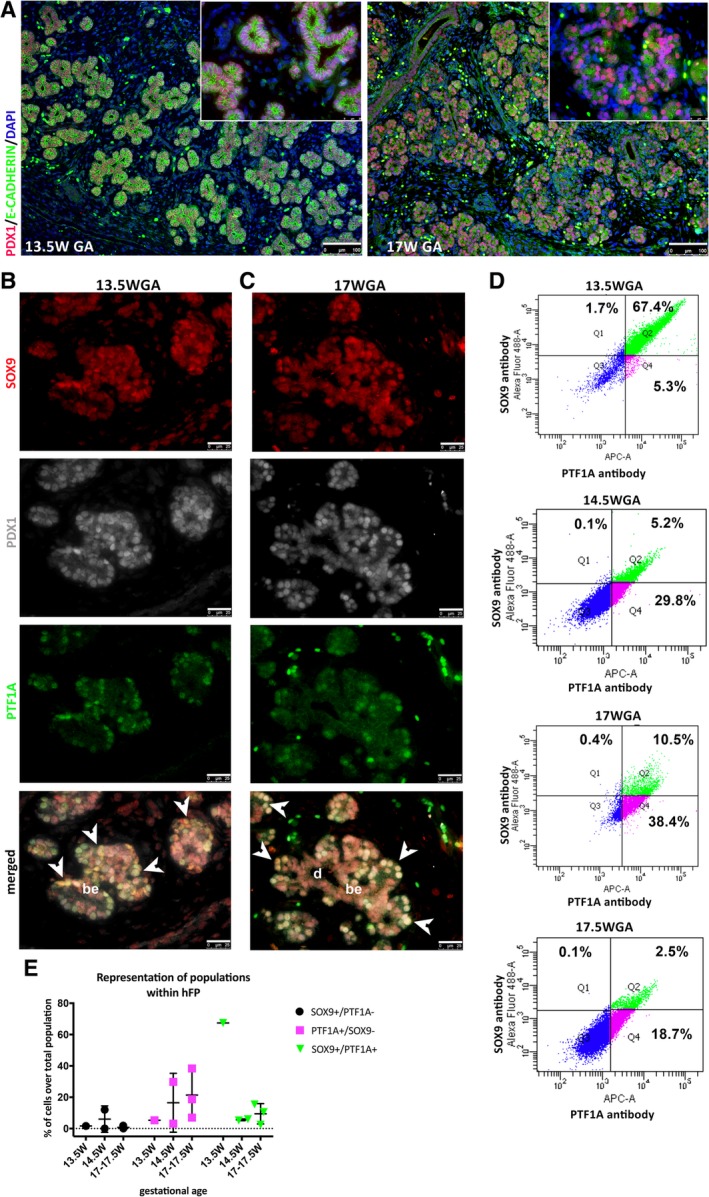
Identification of cells co‐expressing PDX1, SOX9, and PTF1A. **(A):** E‐CADHERIN (green) and PDX1 (red) expression in the pancreatic epithelium at GA 13.5 and 17WGA. Nuclei are in DAPI (blue). Scale bar: 100 μm. **(B, C):** Cells co‐expressing SOX9 (red), PDX1 (gray), and PTF1A (green), indicated by arrowheads, are found at the tips of the branching epithelium (be) at 13.5WGA (B) and 17WGA (C) and are distinguished from developing ducts (d), marked by SOX9 expression. Scale bar: 25 μm. **(D):** Flow cytometry analysis shows that SOX9+/PTF1A+ cells (Q2) were detected at different GA: 13.5W (*n* = 1), 14.5W (*n* = 2), 17W (*n* = 2), and 17.5W (*n* = 1). Single positive SOX9+/PTF1A− cells (Q1) and PTF1A+/SOX9− cells (Q4) were also detected. The percentage of each subpopulation is reported in the respective representative graphs. Quadrant gating was performed based on unstained control for each experiment. **(E):** Representation of SOX9+/PTF1A−, PTF1A+/SOX9−, and SOX9+/PTF1A+ cell subpopulations (individual plot of the percentage of cells over total pancreatic digestion) at different GA (*n* = 1, 13.5W; *n* = 2, 14W; *n* = 3, 17–17.5W). Reduction of the SOX9+/PTF1A+ pool was detected over time with a concomitant increase of PTF1A+ only cells. SOX9+ cells represented a minor fraction, displaying minimal change among GA.

Differentiated structures such as endocrine islets, ducts, branched epithelium, and portions of stratified epithelium were identifiable by H&E staining (Fig. S2). Immunostaining of E‐Cadherin was used to distinguish the pancreatic epithelium from the surrounding mesenchyme. Among E‐Cadherin‐expressing cells, PDX1 was ubiquitously expressed at both 13.5 and 17WGA (Fig. 1A). Subsequently, we co‐stained cells with PDX1, SOX9, and PTF1A. Cells co‐expressing the three markers (PDX1+/SOX9+/PTF1A+) were located at the tips of the branching epithelium at 13.5WGA (Fig. 1B) and 17WGA (Fig. 1C), as well as at 14 and 17.5WGA (Fig. S3A,B).

### The Percentage of SOX9+/PTF1A+ Cells Decreases Overtime

Given that PDX1 expression was ubiquitous in the epithelium, we focused our attention to the SOX9+/PTF1A+ cells. The spatial and temporal identification of SOX9+/PTF1A+ cells within the human pancreatic epithelium is shown in a schematic diagram in Figure S3C. To determine the prevalence of SOX9+/PTF1A+ cells, we performed flow cytometry analysis on primary cell suspensions of hFP at 13.5W (*n* = 1), 14.5W (*n* = 2), 17W (*n* = 2), and 17.5W (*n* = 1). SOX9+/PTF1A+ cells were detected at all GA (Fig. 1D). Plotting the percentage of each subpopulation (SOX9+/PTF1A−, PTF1A+/SOX9−, SOX9+/PTF1A+) across GA showed that at earlier GA there was a higher prevalence (over 60%) of SOX9+/PTF1A+ cells, which decreased at later GA to less than 20% (Fig. 1E). In particular, we observed a large drop in the percentage of SOX9+/PTF1A+ cells between 13.5 and 14.5WGA. The opposite was true for PTF1A+/SOX9− cells, which increased during the same time frame. SOX9+/PTF1A− cells constituted a small population with a constant percentage of cells at all GA.

### SOX9+/PTF1A+ Cells Do Not Express Acinar or Endocrine Proteins

**Figure 2:**
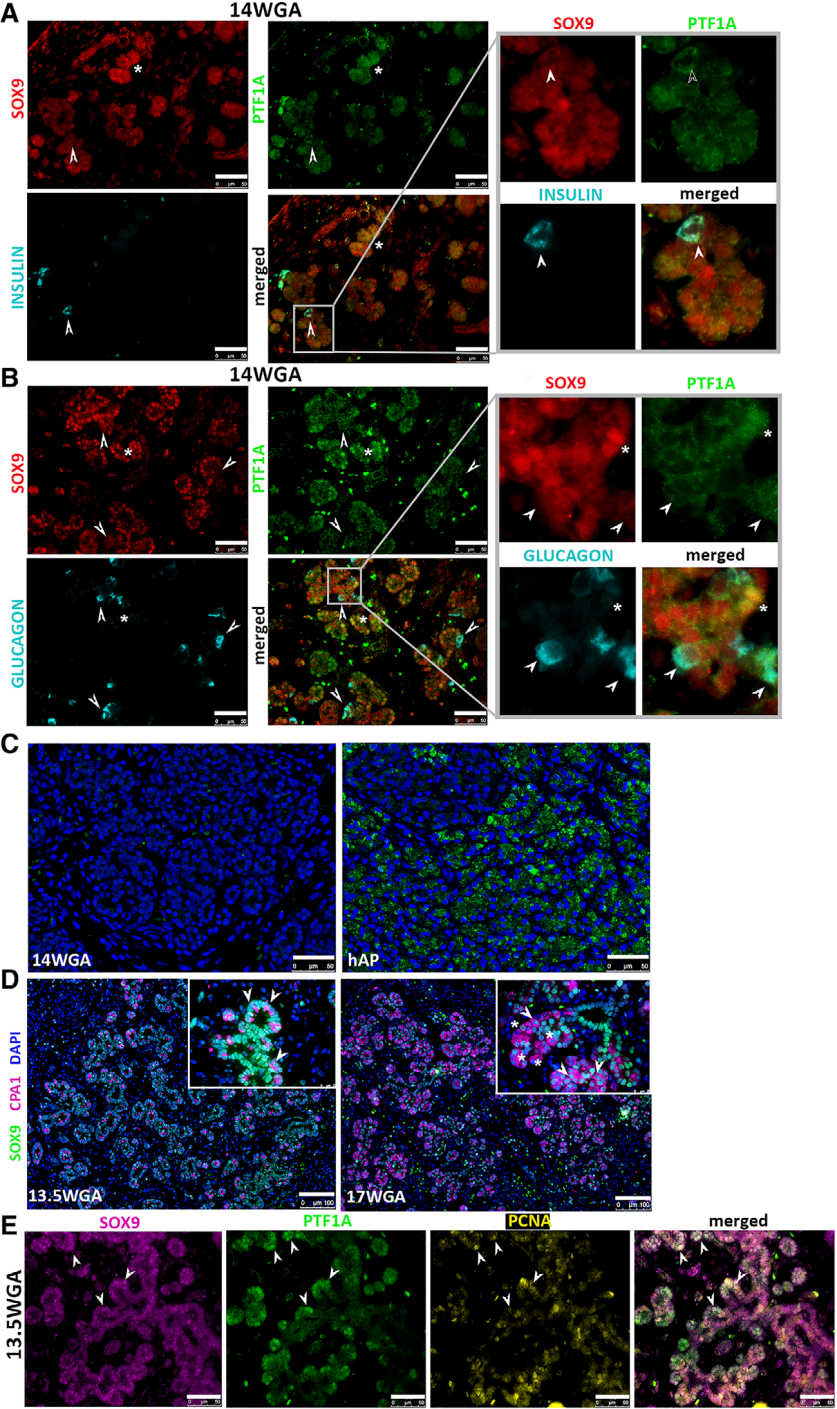
Characterization of SOX9+/PTF1A+ cells. SOX9+/PTF1A+ cells do not express markers of endocrine differentiation as shown by co‐staining with SOX9 (red), PTF1A (green), and either INSULIN (light blue) **(A)** or GLUCAGON (light blue) **(B)**. Scale bar: 25 μm. Magnification of the quadrants indicated in the merged left panels clearly shows that INSULIN and GLUCAGON do not co‐localize with either SOX9 or PTF1A (arrowheads); SOX9 and PTF1A co‐expressing cells are in yellow (asterisks). hFP: 14WGA. **(C):** AMYLASE (green) expression was not detected at 14WGA (left panel). Human adult pancreas (right panel) was used as a positive control to confirm specificity of the AMYLASE antibody. Nuclei are in DAPI. Scale bar: 50 μm. **(D):** Detection of CPA1 expression at 13.5 and 17WGA. CPA1 staining (magenta, cytoplasmic staining) is coupled with SOX9 staining (green, nuclear staining). CPA1 is detected at 13.5WGA, and its expression is noticeably increased by 17WGA. Although all CPA1+ cells also express SOX9 (arrowheads) at 13.5WGA, by 17WGA some CPA1+ cells have lost SOX9 expression (asterisks), suggesting the presence of more committed cells (CPA1+/SOX9−, asterisks) within the tip population at this later stage. However, CPA1+/SOX9+ cells are still detectable at this later stage. Scale bar: 100 μm. **(E):** Co‐staining for SOX9 (magenta), PTF1A (green), and proliferative cell nuclear antigen (PCNA, yellow) shows that SOX9+/PTF1A+ cells are proliferative. Given that a combination of magenta and green results in white and all markers are nuclear, cells positive for SOX9, PTF1A, and PCNA are visible in yellow. Scale bar: 50 μm, hFP: 13.5WGA.

SOX9+/PTF1A+ cells were further examined for the expression of endocrine (insulin and glucagon) or acinar (amylase) differentiation markers using immunohistological staining. Tip cells did not express insulin (Fig. 2A), glucagon (Fig. 2B), or amylase (Fig. 2C) at 14WGA, consistent with results published by others 32. The effectiveness of the anti‐amylase antibodies was verified by staining human adult pancreatic tissue (Fig. 2C). These results demonstrate that SOX9+/PTF1A+ cells do not express lineage‐specific markers.

To further investigate the phenotype of these cells, we evaluated the expression of carboxypeptidase 1 (CPA1), which is expressed in early murine tip MPCs at e12.5 and later in committed acinar cells at e14 7. At 13.5WGA, CPA1 was expressed in a scattered pattern throughout the epithelium; all CPA1+ cells were also SOX9+ (Fig. 2D, arrowheads). In contrast, at 17WGA, some CPA1+ cells lost SOX9 expression (Fig. 2D, asterisks) while others maintained it (Fig. 2D, arrowheads). These results suggest that patterning of the tip progenitor cells versus committed acinar cells is determined around 17WGA. SOX9+/PTF1A+ tip cells also expressed PCNA, a proliferation marker, demonstrating that they were in a proliferative state (Fig. 2E, arrowheads).

### Validation of the Smartflare Technique

**Figure 3:**
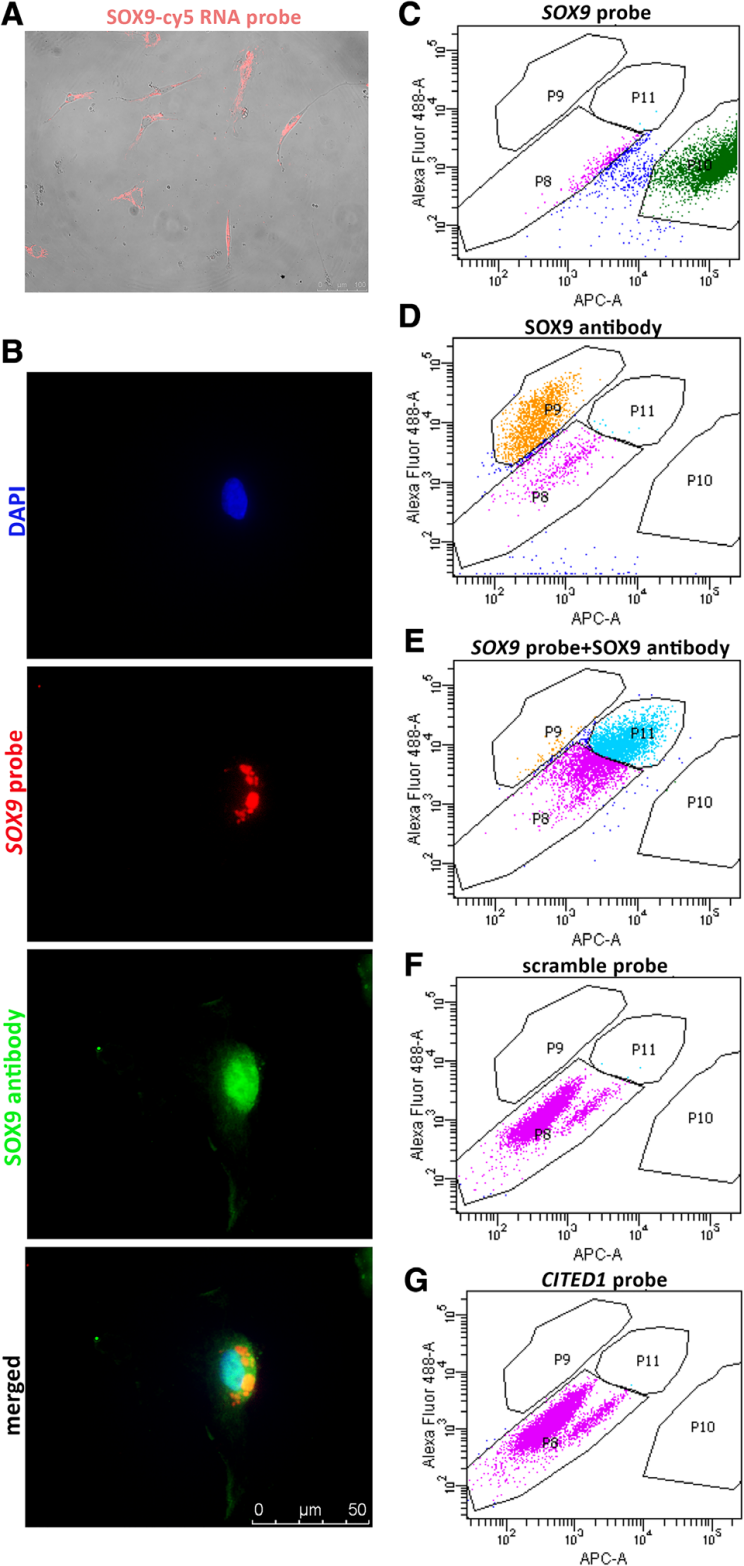
Validation of Smartflare technology. **(A):** hFP tissue (17WGA), dissociated to single cells, was incubated with a *SOX9*‐specific RNA probe. Cells that took up the probes are visualized in red. Scale bar: 100 μm. **(B):** Staining with a SOX9 antibody (green) demonstrated co‐localization with the *SOX9* probe (red) by immunocytochemistry. Nucleus is in DAPI. Scale bar: 50 μm. **(C):** Specificity of the *SOX9* probe validated by flow cytometry analysis. A single cell suspension was treated with the *SOX*9 probe (cy5) (P10). **(D):** Staining with AF488‐conjugated SOX9 antibody (P9). **(E):** Staining with both the *SOX9* probe and the SOX9 antibody, showing separation of co‐stained cells (P11). The antibody detected a minor fraction of cells (P9). To determine efficiency of the probe, we calculated the ratio between the number of cells recognized by both probe and antibody (P11) and the total amount of cells recognized by the antibody (P9 + P11). The *SOX9* probe was 97.5% efficient in detecting the same cells recognized by the SOX9 antibody. Two negative controls were used; **(F)** a scramble cy5‐conjugated probe, and **(G)** a probe targeting transcription factor *CITED1* (not expressed in developing pancreas but expressed in nephrogenic progenitors), further validating specificity of the *SOX9* probe.

Smartflare technology uses RNA probes to recognize RNA targets (including RNAs that code for intracellular proteins and nuclear factors) and can be used to sort live cells 33, 34. A 15WGA hFP was dissociated to a single‐cell suspension and incubated with Cy5‐conjugated *SOX9* RNA probes (Fig. 3A). Cells were then fixed and stained with Alexa Fluor 488‐conjugated SOX9 antibodies. The *SOX9* RNA probe colocalized with SOX9 protein (Fig. 3B), demonstrating that the antibody and the RNA probe identified the same cells. To further validate the technology, we performed flow cytometry analysis using the *SOX9* Smartflare probe and/or an antibody targeting SOX9. The *SOX9* probe alone (Fig. 3C) or the anti‐SOX9 antibody alone (Fig. 3D) stained ~88% or ~71% of the total cell population, respectively. When used in combination, the Smartflare probe and the antibody co‐stained the same population, confirming specificity of the RNA probe (Fig. 3E). We calculated that Smartflare identified 97.5% of the SOX9‐expressing cells based on antibody staining (see legend for Fig. 3). Two negative controls, a scramble probe sequence (Fig. 3F) and a probe for a kidney‐specific transcription factor, *CITED1* 29, did not stain these cells (Fig. 3G). Together, these results validate the specificity of the *SOX9* RNA probe and corroborate the application of this technology for identifying and sorting live cells. Smartflare technology does not affect cell viability, as previously reported 29, 35, 36, 37, 38.

### RNA‐Based Detection of SOX9+/PTF1A+ Cells Confirms their Decrease between 13.5 and 17WGA

To confirm the trends observed through antibody‐based flow cytometry for the percentage of SOX9+/PTF1A−, PTF1A+/SOX9−, and SOX9+/PTF1A+ cells at different GAs (Fig. 1E), we investigated whether a similar pattern could be reproduced using the RNA probes. The percentage of *SOX9*+/*PTF1A*+ cells declined over time, *SOX9*+/*PTF1A*− cells were minimally detected, whereas *PTF1A*+/*SOX9*‐ cells increased with advancing GA (Fig. S4). Together, these results suggest a rapid decline of *SOX9*+/*PTF1A*+ cells over the second trimester.

### A Low Percentage of SOX9+/PTF1A+ Cells Co‐Expressed Cell‐Surface FGFR2

**Figure 4:**
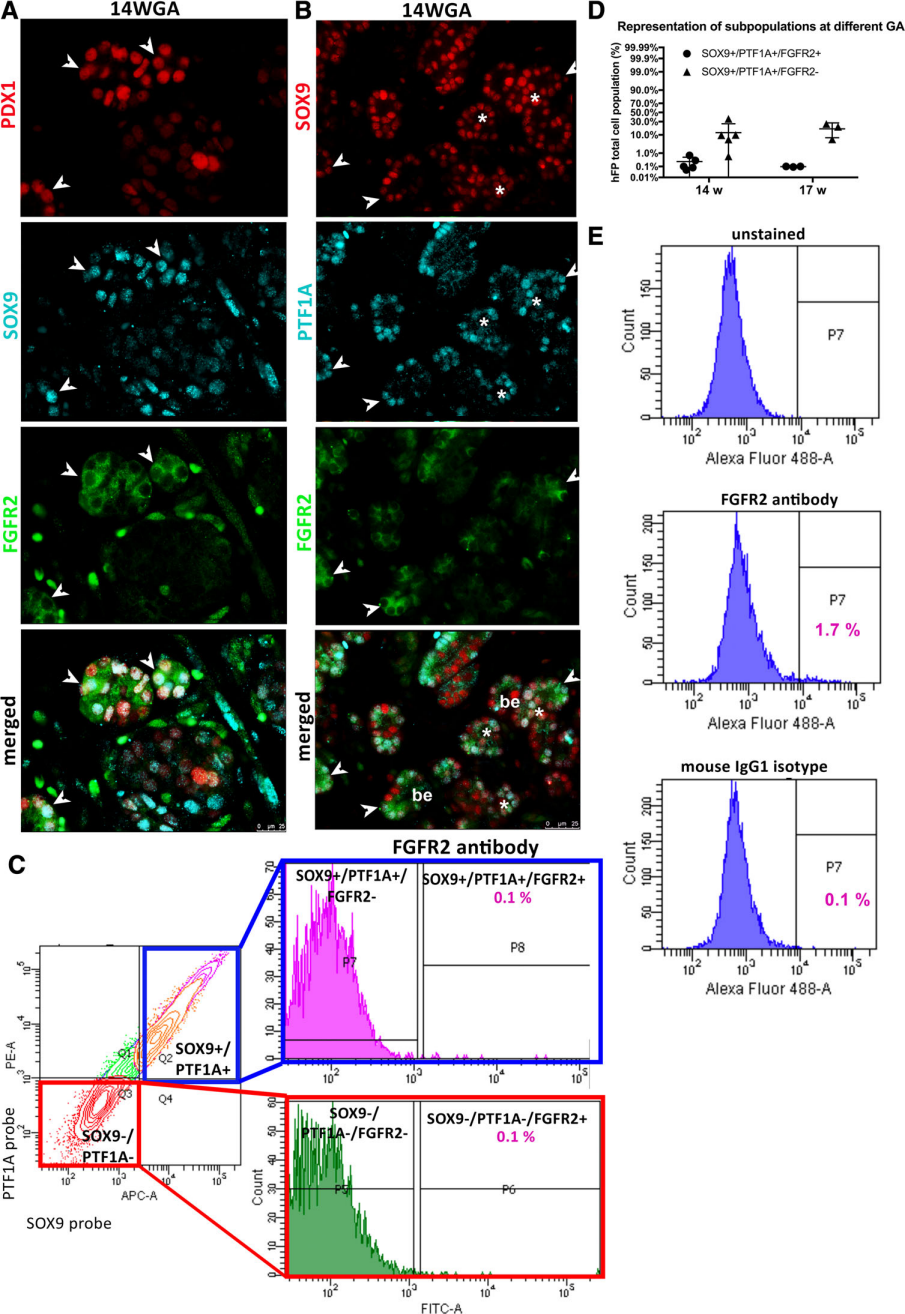
Expression of FGFR2 in hFP. **(A):** Cells co‐expressing PDX1 (red), SOX9 (light blue), and FGFR2 (green) are present at the tips of the branching epithelium (arrowheads). **(B):** Cells co‐expressing SOX9 (red), PTF1A (light blue), and FGFR2 (green) are found at the tips of the pancreatic epithelium (arrowheads). Cells co‐expressing SOX9 and PTF1A, but not expressing FGFR2, are also present at the termini of the branches (asterisks). be = branching epithelium. Scale bar: 25 μm, hFP: 13.5WGA. **(C):** Sorting strategy for the isolation of subpopulations. Cells were gated based on the expression of *SOX9* and *PTF1A*, detected through RNA probes, and gated for the expression of FGFR2. SOX9+/PTF1A+/FGFR2+ (P8) cells represent a small fraction (0.1%) of the total hFP cell population compared to SOX9+/PTF1A+/FGFR2− (P7) cells, which constitute on average 15% of total hFP. The negative fraction of cells (SOX9−/PTF1A−, Q3) was also gated on FGFR2, confirming that cells expressing FGFR2 (SOX9−/PTF1A−/FGFR2+, P6) represent only 0.1%, whereas the majority of SOX9−/PTF1A− cells are also FGFR2− (SOX9−/PTF1A−/FGFR2−, P5). **(D):** Distribution of SOX9+/PTF1A+/FGFR2− and SOX9+/PTF1A+/FGFR2+ subpopulations at 14 and 17WGA. **(E):** Given the very low expression of FGFR2, specificity of the FGFR2 antibody was tested by using a mouse IgG1 isotype control on total pancreatic digestion. In order are: an unstained control, a sample stained with FGFR2 antibody (staining 1.7% of the cells), and a sample stained with a mouse IgG1 isotype (showing 0.1% of nonspecific signal).

Fibroblast growth factor (Fgf) signaling creates a feed‐forward loop with Sox9 to maintain pancreatic organ identity during pancreas development in mice 5. We examined the expression pattern of FGF receptor, FGFR2, which is known to bind FGF1, 7, and 10 2. As demonstrated by triple immunostaining, some tip cells that were SOX9+/PDX1+ or SOX9+/PTF1A+ expressed FGFR2 at 14WGA (Fig. 4A‐B). Further analysis within the pancreatic epithelium at different GA (13.5, 14.5, 17, and 17.5W) revealed the presence of SOX9+/PTF1A+/FGFR2+ cells (arrowheads), but SOX9+/PTF1A+/FGFR2− cells (asterisks) were also present (Fig. S5).

To quantify the percentage of SOX9+/PTF1A+/FGFR2+ cells, flow cytometry analysis of live cells was performed. hFPs (13.5–14.5WGA, *n* = 4 and 16.5–17.5WGA, *n* = 3) were dissociated into single‐cell suspensions, incubated with *SOX9* and *PTF1A* Smartflare RNA probes and then stained with antibodies against cell‐surface protein FGFR2. We first gated on SOX9+/PTF1A+ double‐positive cells (Fig. 4C; left panel), which accounted for an average of 15%–17% among the total cells at different GAs (not shown). The SOX9+/PTF1A+ cells were further gated on FGFR2 (Fig. 4C; right upper panel). SOX9+/PTF1A+/FGFR2+ cells were a small population, constituting 0.1%–0.4%, while SOX9+/PTF1A+/FGFR2− cells accounted for 99.9% of the SOX9+/PTF1A+ fraction (Fig. 4C). Little variability was observed in the percentage of SOX9+/PTF1A+/FGFR2+ cells among the total dissociated cells between 14 and 17WGA (Fig. 4D). The gated SOX9‐/PTF1A− cells were also negative for the expression of FGFR2 (Fig. 4C). The low percentage of FGFR2+ cells in the gated SOX9+/PTF1A+ cells was not due to ineffective antibodies; FGFR2 stained 1.7% of the total un‐gated population in a 17WGA sample, whereas a mouse IgG1 isotype control showed background staining of 0.1% (Fig. 4E). To further confirm, anti‐FGFR2 antibodies stained 5.3% positive in primary human fetal lung cells (Fig. S5E), which were used as positive control. Together, these results demonstrate that the majority of the SOX9+/PTF1A+ cells do not express FGFR2, at least at the cell‐surface. We speculate that SOX9+/PTF1A+ cells may use other FGFRs, such as FGFR1, 3, and 4, for signaling. Alternatively, human SOX9 may function differently compared to the murine gene in inducing Fgfr2 expression 5. Given the very low expression of cell surface FGFR2, we focused only on SOX9+/PTF1A+/FGFR2− cells for subsequent analysis.

### SOX9+/PTF1A+ Cells Maintain a Similar Gene Expression Profile over Developmental Time while SOX9−/PTF1A− Cells Diverge

**Figure 5:**
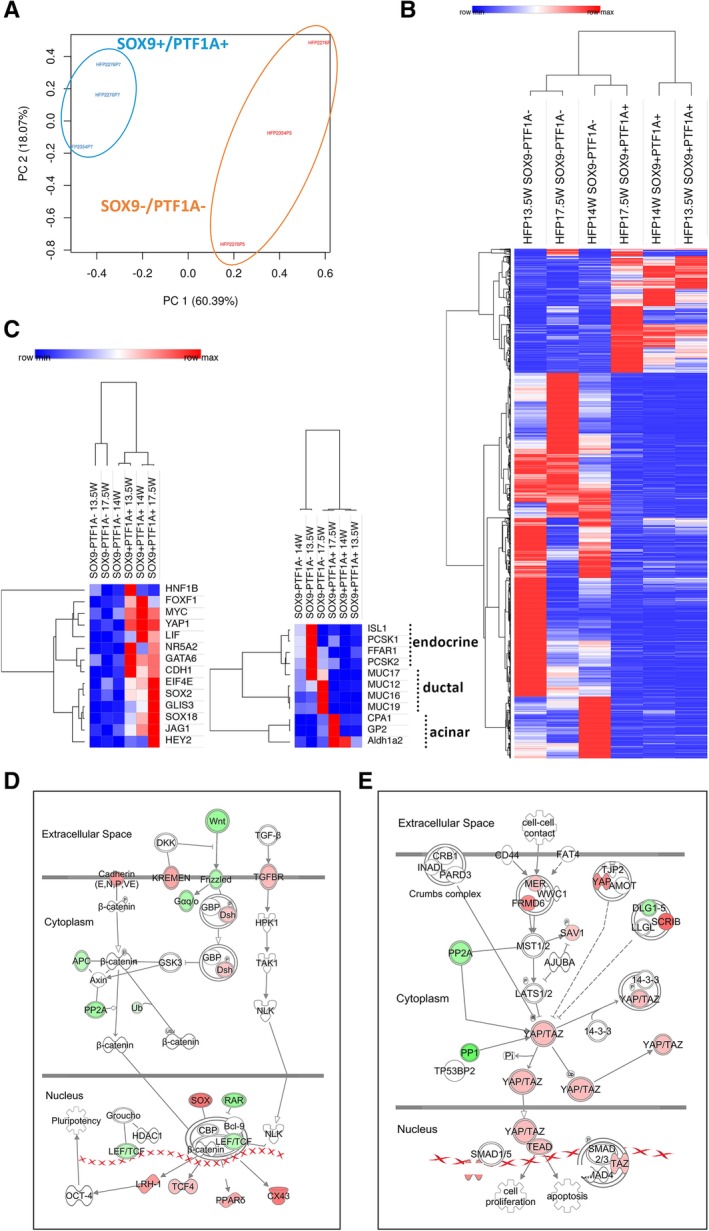
RNA‐sequencing profiling of *SOX9*+/*PTF1A*+ cells. **(A):** Principal component analysis shows that SOX9+/PTF1A+ populations from *n* = 3 different hFP samples (respectively, 13.5, 14, and 17.5WGA) cluster together. Negative fractions for the respective isolated populations cluster independently. **(B):** Heatmap representation of overall gene expression (*p* < .05, FDR *p* < .05) for SOX9+/PTF1A+ and SOX9−/PTF1A− cell populations at the indicated GA. Hierarchical clustering shows similarity among the SOX9+/PTF1A+ populations, particularly at 13.5 and 14WGA, with a slight change at the later GA (17.5W). Negative fractions display more variability in terms of overall gene expression. **(C):** Heat maps of gene expression of markers characteristic of multipotent progenitors (left) and markers of differentiation including endocrine, ductal, and acinar lineages (right). Gene list for multipotent markers: *HNF1B*, *FOXF1*, *MYC*, *YAP1*, *LIF*, *NR5A2*, *GATA6*, *CDH1*, *EIF4E*, *SOX2*, *GLIS3*, *SOX18*, *JAG1*, and *HEY2*. Gene list for differentiation markers: *ISL1*, *PCSK1*, *FFAR1*, *PCSK2*, *MUC17*, *MUC12*, *MUC16*, *MUC19*, *CPA1*, *GP2*, and *ALDH1A2*. Values are mapped to colors using the minimum (blue) and maximum (red) of each row (gene) independently. IPA analysis of the Wnt/ß‐catenin pathway **(D)** and the Hippo pathway **(E)** showing differentially expressed genes between SOX9+/PTF1A+ and the negative counterpart. Upregulated genes are in red, downregulated genes are in green. Genes represented in white were nonsignificant and therefore not detected by IPA analysis. Filters applied for the analysis: *p* < .05, FDR *p* < .05, and logFC < −1.5 or >1.5.

Given the ability to sort live cells, we performed bulk RNA‐seq analysis to determine the global gene expression profiles of SOX9+/PTF1A+ cells and SOX9−/PTF1A− cells from *n* = 3 hFP samples (13.5, 14, and 17.5WGA). Principal component analysis showed that SOX9+/PTF1A+ populations clustered closer together compared to SOX9−/PTF1A− populations (Fig. 5A). This result was further corroborated by the hierarchical clustering analysis (one minus Pearson correlation) of overall gene expression (*p* < .05, FDR *p* < .05) (Fig. 5B), which showed that SOX9+/PTF1A+ cell populations at 13.5 and 14WGA clustered together, whereas a change with a slight increase in gene expression was appreciated at 17.5WGA. In contrast, SOX9−/PTF1A− fractions displayed more divergence in expression patterns. This result is consistent with the drop in the percentage of SOX9+/PTF1A+ cells over time (Fig. 1E and Fig. S4), and suggests that during the second trimester more rapid changes were happening within the SOX9−/PTF1A− fractions, which include cells committed to not only endocrine, ductal, and acinar fate but also other cell types of the endothelial and mesenchymal lineages. We next analyzed the expression of markers that are characteristic of either MPC identity or lineage differentiation. SOX9+/PTF1A+ cells displayed enrichment for genes expressed by multipotent progenitor cells compared to SOX9‐/PTF1A‐ cells. Genes, such as the transcription factors *MYC*, *YAP1*, and *GATA6*, as well as *CDH1* (*E‐Cadherin*), were enriched in all three SOX9+/PTF1A+ populations, regardless of the GA analyzed (Fig. 5C, left panel). As expected, expression of endocrine and ductal differentiation markers showed an opposite trend (Fig. 5C, right panel) and was downregulated in SOX9+/PTF1A+ cells. However, the typical exocrine cell markers, *CPA1* and *GP2* for acinar and *ALDH1A2* for centro‐acinar cells, were enriched only in the SOX9+/PTF1A+ cells isolated from the later but not earlier GAs (Fig. 5C, right panel). These results are consistent with the lack of committed acinar cells in the 14WGA hFP (Fig. 2C), and suggest that *CPA1*, *GP2*, and *ALDH1A2* mark progenitor cells instead during the second trimester.

### WNT and YAP Pathways Are Inactive and Active, Respectively, in the Sorted SOX9+/PTF1A+ Versus SOX9−/PTF1A− Cells

Multiple signaling pathways are known to govern progenitor cell proliferation and cell‐fate determination in the developing pancreas 39, 40, 41, 42, 43, 44. To determine which signaling pathways were most significant in the SOX9+/PTF1A+ cells, we used a bioinformatics tool, IPA. Components in the Wnt/β‐catenin (Fig. 5D) and the Hippo signaling (Fig. 5E) emerged as primarily inactive (green) and active (red), respectively, in the SOX9+/PTF1A+ cells. In mice, knockout of Hippo signaling in the whole pancreas causes defects in exocrine but not endocrine development 44, 45, suggesting MPCs are not affected. In contrast, Wnt signaling has a major effect on MPC maintenance in murine fetal pancreas 46. Hippo is known to regulate the expansion of pancreatic MPCs derived from embryonic stem cells 47; however, whether Hippo plays a functional role in human fetal MPCs remained to be established.

This result suggests that Hippo but not Wnt signaling may be the dominant pathway governing MPC functions at the second trimester.

### Gene Set Enrichment Analysis (GSEA) Reveals Active Branching Morphogenesis of the SOX9+/PTF1A+ Cells

**Figure 6:**
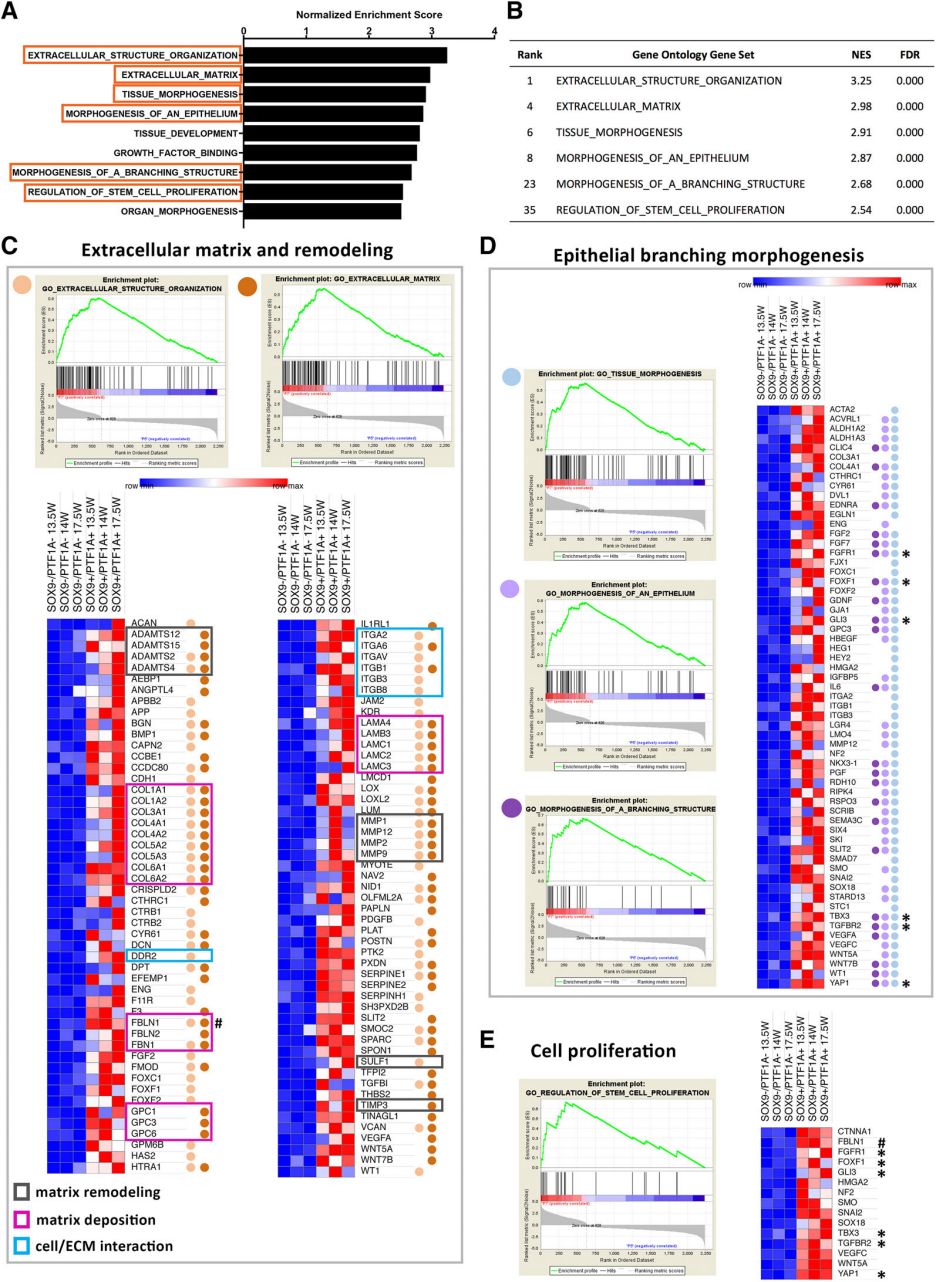
GSEA of gene expression profiling of *SOX9*+/*PTF1A*+ cells. **(A):** Enriched GO gene sets within SOX9+/PTF1A+ cells belong mostly to ECM processes and tissue development/morphogenesis. **(B):** List of selected GO gene sets relevant to pancreas development for leading edge analysis, with their respective ranking and normalized enrichment score (NES). FDR, false discovery rate. **(C):** GO enrichment plots for extracellular matrix deposition and remodeling related gene sets, including GO for extracellular structure organization and extracellular matrix (top). Heat map representation of leading edge analysis for the two gene sets (bottom). Overlap between leading edge subsets is marked by color‐coded dots. Genes with specific functions related to ECM processes have been highlighted to include three main groups: matrix remodeling, matrix deposition, and cell/ECM interaction. **(D):** GO enrichment plots for epithelial branching morphogenesis‐related gene sets, including GO for tissue morphogenesis, morphogenesis of an epithelium, and morphogenesis of a branching structure (left). Heat map representation of leading edge analysis for the three gene sets (right). Overlap between leading edge subsets is marked by color‐coded dots. **(E):** GO enrichment plot of GO for regulation of stem cell proliferation and heat map representation of gene expression.

To corroborate our hypothesis and further characterize the SOX9+/PTF1A+ cells, we performed GSEA. Among the top‐50 GO (Gene Ontology) sets, the extracellular matrix (ECM), tissue development and morphogenesis, and stem‐cell proliferation emerged as major processes, as shown by their higher normalized enrichment scores (Fig. 6A). The rankings of six selected gene sets were further listed (Fig. 6B). Next, we performed leading‐edge analysis to visualize the genes that were significantly enriched in the SOX9+/PTF1A+ cells, with regards to the ECM remodeling (Fig. 6C), epithelial branching morphogenesis (Fig. 6D), and cell proliferation (Fig. 6E). A few hubs of genes of interest were identified accordingly. In the GOs related to ECM, we noted genes for matrix remodeling (such as *ADAM* and *MMP* family genes) (Fig. 6C; black boxes), matrix deposition (such as *COL*, *GPC*, *FB*, and *LAM* family genes) (Fig. 6C; red boxes), and cell/ECM interactions (*DDR* and *ITG* family) (Fig. 6C; blue boxes). In the GOs related to epithelial branching morphogenesis (Fig. 6D), various genes related to growth signals (Fig. 6D, asterisks) and cell/ECM interactions were also enriched. Finally, in the GO of cell proliferation, a matrix deposition gene (*FBLN1*) appeared again (Fig. 6E), as well as genes (*FGFR1*, *FOXF1*, *GLI3*, *TBX3*, *TGFBR2*, and *YAP1*) that were significantly represented in the GO of epithelial branching morphogenesis (Fig. 6D,E, asterisks). Taken together, these results suggest intimate relationships between branching morphogenesis, ECM remodeling and cell proliferation in the SOX9+/PTF1A+ cells.

### Single‐Cell (sc) RNA‐Sequencing of a Human Pancreas of 15.4WGA

**Figure 7:**
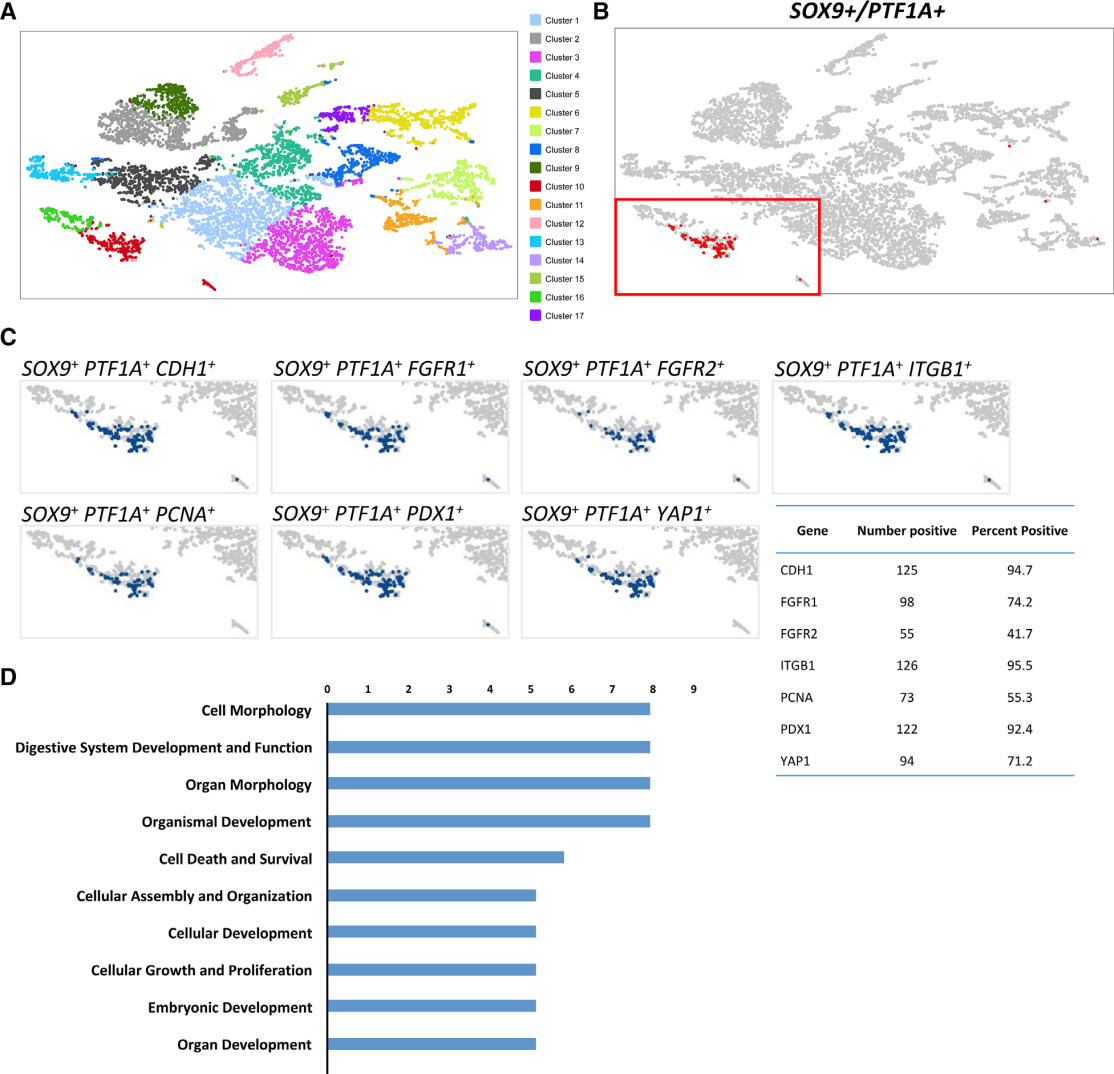
Single‐cell RNA‐seq of a 15.4WGA hFP. **(A):** tSNE plot of single‐cell RNA‐seq of ~8,000 cells dissociated from a 15.4 WGA hFP. Seventeen clusters were identified. **(B):** Identification of SOX9+/PTF1A+ cells within a single cluster (cluster #10, red). **(C):** Visualization of SOX9+/PTF1A+ cells (within cluster #10) and expression of different progenitor markers and their relative representation within the cluster. Genes listed: *CDH1*, *CTNNB1*, *FGFR1*, *FGFR2*, *ITGB1*, *PCNA*, *PDX1*, and *YAP1*. **(D):** IPA analysis of cluster 10 obtained from the tSNE analysis. Differentially expressed genes (*p* < .05, FDR *p* < .05) were used to identify the most significant biological functions for cluster #10 where SOX9+/PTF1A+ cells were identified. The top 10 most relevant functions are listed here.

To further dissect the developmentally‐regulated processes of the hMPCs, we performed scRNA‐seq (hFP, 15.4WGA). This GA comes after the reduction in the % SOX9+/PTF1A+ cells that was observed and therefore more representative of the MPCs of the second trimester (Fig. 1E). A total of 17 different clusters were identified and visualized in a t‐Distributed Stochastic Neighbor Embedding (tSNE) plot, which reduces the highly complex gene expression patterns of the individual cells into a two‐dimensional plot (Fig. 7A).

The vast majority of SOX9+/PTF1A+ cells were located in cluster #10 (Fig. 7B). SOX9+/PTF1A+ cells expressed *CDH1*, *PCNA*, and *PDX1* (Fig. 7C), consistent with the findings from the immunohistological staining (Figs. 1 and 2). The fact that 92.4% of SOX9+/PTF1A+ cells expressed *PDX1* confirmed a MPC‐like phenotype. Consistent with the bulk RNA‐seq analysis (Fig. 6C), the majority (95.5%) of SOX9+/PTF1A+ cells expressed *ITGB1*, suggesting a cell‐matrix interaction. *YAP1* and *PCNA* were expressed in 71.2% and 55.3%, respectively, of SOX9+/PTF1A+ cells, suggesting a subpopulation is proliferating. These results are largely consistent with the results from the bulk RNA‐sequencing of *n* = 3 different SOX9+/PTF1A+ donor populations (Fig. 6C–E). The percentage of SOX9+/PTF1A+ cells co‐expressing *FGFR2* was lower than *FGFR1* (41.7% vs. 74.2%, respectively); consistent with the finding that FGFR2 was minimally expressed on the cell surface (Fig. 4).

To determine whether the abovementioned markers were expressed beyond cluster #10, we removed the filter on SOX9+/PTF1A+ cells and examined the gene expression patterns in all clusters (Fig. S6). Except for *ITGB1*, *PCNA*, and *FGFR1*, which were expressed in other clusters, *SOX9*, *PTF1A*, *CDH1*, *FGFR2*, and *YAP1* were mostly exclusively represented in cluster #10. *PDX1* was enriched in both cluster #10 and cluster #12, which identifies the endocrine cluster (Fig. S6), confirming its role in both MPCs and endocrine lineage. This result strengthens our findings from bulk RNA‐seq.

Next, we investigated the expression of various lineage markers (Fig. S7). The mesenchyme marker *VIM* was expressed in the majority of the clusters at this developmental stage (Fig. S7A), whereas the marker for nascent endothelium (*TIE1*) was found only in cluster #6 (Fig. S7B). Cluster #12 was enriched for committed endocrine cells, as shown by *GCG* expression (Fig. S7C). Consistent with prior reports 22, 24, 48, 49, *PDX1*, *NKX6.1*, and *GATA6* were expressed in both MPC‐like (#12) and endocrine (#10) cells (Fig. S7D–F). Compared to *PDX1*, NGN3 was expressed in fewer cells in the endocrine and the MPC clusters (Fig. S7G), supporting the idea that NGN3 is expressed transiently before commitment from progenitor cells into endocrine cells. Consistent with the lack of acinar cells (Fig. 2C), *AMY2A* was not detected (Fig. S7H) and *AMY2B* was minimally expressed (Fig. S7I). Interestingly, a pluripotent stem cell marker, NANOG, was expressed mostly in the MPC cluster (#10, Fig. S7J)—the significance of this finding is unknown, but NANOG has been associated with the formation of pancreatic ductal adenocarcinoma 50.

To further identify potential functional pathways within the cluster containing SOX9+/PTF1A+ cells, we analyzed the genes that were differentially expressed between cluster #10 and the other clusters combined, using IPA analysis. The most significant processes are listed in Figure 7D, which included cell morphology, digestive system development and function, organ morphology, organismal development, cell death and survival, cellular assembly and organization, cellular development, cellular growth and proliferation, embryonic development, and organ development. Genes identified in each of these biological functions are listed in Figure S8. Together, these results confirm the bulk RNAseq data and demonstrate that SOX9+/PTF1A+ cells of the 15.4WGA are active in organ development, morphogenesis and proliferation.

## Discussion

Several mouse models have proved useful to elucidate the mechanisms involved in the maintenance of tip MPCs and their lineage commitment during pancreatic development 2, 51. Studies of the human pancreas revealed similarities between murine and human systems, including the expression of crucial transcription factors. However, important differences with regard to the timing and expression patterns of some of these factors were found 22, 32. Few studies reported on the development of the human pancreas in the second trimester and mostly focused on endocrine differentiation 52, 53. A recent work on the single‐cell profiling of the hFP at 9WGA showed that distinct populations of progenitor cells and endocrine cells, each containing subpopulations of maturation intermediates, can be found at a single time point during development 54.

Information on human MPCs residing within the hFP has been limited. Although some studies have investigated the early embryonic stage of human pancreas development 24, others have mainly focused on the characterization of SOX9+ trunk progenitor cells. The spatial and temporal expression pattern of SOX9 during human pancreas development was dissected, with an emphasis on its role in islet cell differentiation 53. However, the role of PTF1A in the hFP has been elusive. Our current studies build on the prior knowledge and show that SOX9+/PTF1A+ cells are found at the tips of the pancreatic branching epithelium, which is a different population than the previously characterized trunk progenitor cells.

A technological significance of our study is the application of RNA probes 33, 34 to isolate live progenitor cells from human fetal pancreas. This method has proven valid in other cell types 35, 36, 37, 38, and using this technology we have successfully isolated renal progenitor cells in a prior report 29. Here, we isolated pancreatic cells expressing *SOX9* and *PTF1A* transcripts, demonstrating a high correlation (97.5%) between the probe and the antibody detection of the same marker. However, it should be noted that Smartflare probes are not ideal for the quantitative analysis of subtle mRNA levels due to a narrow dynamic range of detection 55. Nevertheless, our data validate the qualitative use of Smartflare probes to identify cells expressing SOX9 and PTF1A and study their genetic profile.

All of the three main pancreatic lineages, including endocrine, ductal and acinar, originate from an initial stratified epithelium containing the primordial MPCs. Once the process of branching morphogenesis begins, progenitor cells can be discriminated into trunk and tip progenitor cells, based on their location within the epithelium. The trunk progenitor cells eventually give rise to ductal and endocrine cells, whereas the MPC domain, located at the branching tips, maintains multipotency until becoming restricted to the acinar lineage. This switch of the tip multipotent progenitor cells to the acinar lineage coincides with the “second wave” of the endocrine pancreas development in mice 7, 56. The first wave is characterized by the presence of a few Ngn3+ endocrine progenitor cells and endocrine cells that co‐express multiple hormones. In the second wave which starts around e12.5, an upregulation of the Ngn3+ endocrine progenitor cells is followed by the appearance of endocrine cells that express only single hormones. However, in the human pancreas a single‐phase of NGN3 expression and endocrine commitment was detected starting at 8WGA and onward 24. This further highlights the differences between the murine and the human pancreas development.

Information regarding the occurrence and timing of a developmental switch of the tip MPCs during human development is limited 57. The proportion of SOX9+/PTF1A+ cells among the total cells in the hFP at 13.5WGA was higher than the proportion we observed at 14–17WGA. Concomitantly, the proportion of PTF1A+/SOX9− cells increased. Thus, the loss of SOX9 expression and the maintenance of PTF1A suggest a commitment of the SOX9+/PTF1A+ cells toward acinar lineage 58. This was further corroborated by comparing SOX9 and CPA1 expression between earlier and later GA. Cpa1 was confirmed as MPC marker through lineage tracing studies 7. Cpa1, Pdx1, Ptf1a, and c‐Myc mark the murine tip MPC domain and these cells are multipotent before becoming restricted to the acinar lineage at mid‐gestation around e14 7. Here, we observed that all CPA1+ cells were also SOX9+ at 13.5WGA, whereas at later GA, some of the CPA1+ cells were not SOX9+ (Fig. 2D), suggesting that patterning of the tip cells occurred in the hFP between 13.5 and 17WGA. However, contrary to a prior report 59, we did not observe expression of amylase at the protein level in any specimen (Fig. 2C). Similarly, mRNAs for *AMY2A* and *AMY2B* were largely absent in the scRNA‐seq analysis. These results confirm amylase as a late differentiation marker for the acinar lineage.

Bulk RNA‐sequencing profiling confirmed that *SOX9*+/*PTF1A*+ cells from *n* = 3 donor tissues clustered together, revealing similarities on overall gene expression; in contrast, the negative fractions displayed greater divergence. This result suggests that more rapid changes occur during developmental time within the non‐MPC fractions of the hFP, which include committed or differentiating cells, whereas the *SOX9*+/*PTF1A*+ fraction preserves its identity/gene expression profile (Fig. 5B). Interestingly, we found *GATA6* enriched in the SOX9+/PTF1A+ cells (Figs. 5C and 7C). Contrary to what has been observed in the mouse 60, mutations of the human *GATA6* gene have been associated with pancreatic agenesis 61, 62, 63, consistent with *GATA6* as a MPC marker in human. A recent work also confirmed the role of *GATA6* as a regulator of definitive endoderm (DE) and early pancreatic morphogenesis in *GATA6*‐null human pluripotent stem cells, which failed to enter the DE program upon induction to differentiate 64.

Molecular signaling pathways such as Notch, FGF, and WNT/β‐catenin are known to play critical roles in determining specification of nonhuman mammalian pancreas and regulating endocrine/exocrine differentiation 5, 40, 41, 43. Surprisingly, these pathways did not appear to play significant roles in the SOX9+/PTF1A+ cells, using various bioinformatics analyses. We, instead, found the Hippo pathway being relevant in the SOX9+/PTF1A+ cells. The Hippo pathway is a universally conserved pathway involved in regulating organ size growth during development and controls the fine balance between pluripotent stem cells and lineage‐restricted cell pools in mammals 65. Evidence indicates that YAP, which is the main nuclear target of Hippo signaling and is negatively regulated by it, is highly expressed by the stem or progenitor cell niche of different adult epithelial tissues, including the intestinal crypts and the skin in both mice and humans 66, 67. The Hippo signaling pathway acts during the second wave in mice to determine proper architecture of the pancreas but not endocrine differentiation 44. More recent studies demonstrate that the YAP/TEAD complex functions during early pancreas development to expand human MPCs, and is subsequently lost during lineage differentiation 47. Inhibition of YAP results in enhanced differentiation of functional ß‐cells from hPSCs, whereas a sustained in vitro activation of YAP results in impaired ß‐cell differentiation 68. Thus, our finding on the *YAP*/*TEAD* complex being upregulated in the SOX9+/PTF1A+ cells isolated from the second trimester (Fig. 5E) is consistent with the idea that these cells are the uncommitted MPC‐like cells with potential to direct organ size and architecture.

ECM deposition and remodeling play a crucial role during tissue development and epithelial morphogenesis, when progenitor cells constantly degrade old matrixes and deposit new ones to allow cell expansion and organ growth 69. From the GSEA analysis, we indeed found SOX9+/PTF1A+ cells enriched for expression of genes involved in matrix remodeling and cell proliferation, suggesting active branching and epithelium expansion. ECM remodeling proteins regulate matrix degradation and allow progenitor cells to expand 70. We found ECM remodeling genes, including family proteins of metalloproteinases and a disintegrin and metalloproteinase with thrombospondin motif (ADAMTS), were upregulated in SOX9+/PTF1A+ cells, compared to SOX9−/PTF1A− cells (Fig. 6B). Integrin‐mediated ECM‐cell interaction has long been recognized for its importance in tissue development and maintenance of progenitor cells. We observed upregulation of *ITGB1*, consistent with previous findings in the hFP 71, 72, and *ITGA6*, which may bind to *ITGB1*, was reported as a marker for the isolation of progenitor cells from the human fetal pancreas 73. In addition, we detected enrichment of *ITGAV* 74, *ITGA2* and *ITGB3* at all GAs analyzed, which have not been previously reported as putative markers of human MPCs.

FGF signaling is known to promote the proliferation of murine pancreatic progenitor cells via an Fgfr2/Sox9 regulatory axis. However, we detected very few SOX9+/PTF1A+ cells expressing FGFR2 protein at the cell surface (~1%, Fig. 4), despite the fact that approximately 40% of these cells express the *FGFR2* transcripts, as detected in the scRAN‐seq analysis (Fig. 7). Interestingly, *FGFR1* transcript was enriched in the *SOX*+/*PTF1A*+ population (Fig. 7A) together with *FGF7* and *FGF2* (Fig. 6D). FGF2 is a direct ligand for FGFR1. The isoform IIIb of the FGFR1 was reported to be exclusively expressed in the rat pancreatic epithelium and as such proposed as a potential marker of pancreatic progenitor cells 75. Inhibition of FGFR1 was shown to promote the terminal differentiation of human induced pluripotent stem cell (hiPSC)‐derived endocrine progenitor cells into endocrine cells 76. Therefore, FGFR1, rather than FGFR2, could be a more significant player of the FGF signaling in human pancreas development. Further investigation will be needed to validate this hypothesis.

## Conclusion

We have demonstrated the presence of human MPC‐like cells expressing SOX9 and PTF1A at the tips of the branching epithelium of the developing pancreas during the second trimester. Multiple gene‐expression analyses of SOX9+/PTF1A+ cells indicate that these cells retain the profile of MPCs, including expression of known key transcription factors, proliferation, branching, and signaling pathways. Some newly‐identified candidate genes, such as *FGFR1*, *TGFBR2*, *FOXF1*, *GLI3*, *TBX3*, as well as *NANOG*, *ITGAV*, *ITGA2*, and *ITGB3*, have not been previously described in either murine or human fetal pancreatic progenitor cells, making them suitable candidate for screening and enrichment of in vitro‐derived human pancreatic progenitor cells. Mechanosignaling via integrin molecules might play a crucial role in maintaining the human pancreatic MPC niche. Further investigations into the functions of hMPCs are needed and are currently in progress in our laboratories.

In summary, the results we have described here provide a better understanding of the molecular programs that sustain pancreatic progenitor cells during the second trimester of human development. Although many aspects of the progenitor‐cell molecular signature are conserved between mice and humans, we have identified some key differences that may provide insight in developing better protocols for differentiation of human pluripotent cells into the pancreatic lineages. Ultimately, these efforts may lead to therapeutically relevant cells such as beta cells that might be used for cell replacement therapy for type 1 diabetes.

## Author Contributions

V.V.: conception and design, collection and/or assembly of data, data analysis and interpretation, manuscript writing; M.E.T.: provision of study material, data analysis and interpretation; H.N.Z. and C.C.: data analysis and interpretation; B.H.G.: provision of study material, manuscript writing; G.O.: data analysis and manuscript writing; R.D.F.: data analysis and interpretation; H.T.K.: conception and design, data analysis, manuscript writing; L.P.: conception and design, data analysis, final approval of manuscript.

## Disclosure of Potential Conflicts of Interest

The authors declare no competing or financial interests.

## Supporting information


**Appendix S1**: Supporting Information

## Data Availability

The data that support the findings of this study are available from the corresponding author upon reasonable request.
