# Supplementary material for: SOX9+/PTF1A+ Cells Define the Tip Progenitor Cells of the Human Fetal Pancreas of the Second Trimester
Source: Stem Cells Transl Med. 2019 Oct 21;8(12):1249–64. doi: 10.1002/sctm.19-0231 (PMC6877773; doi:10.1002/sctm.19-0231)
Supplement: Supplementary file 1 — Appendix S1: Supporting Information [file SCT3-8-1249-s001.pdf]

# Supplementary Figure 1

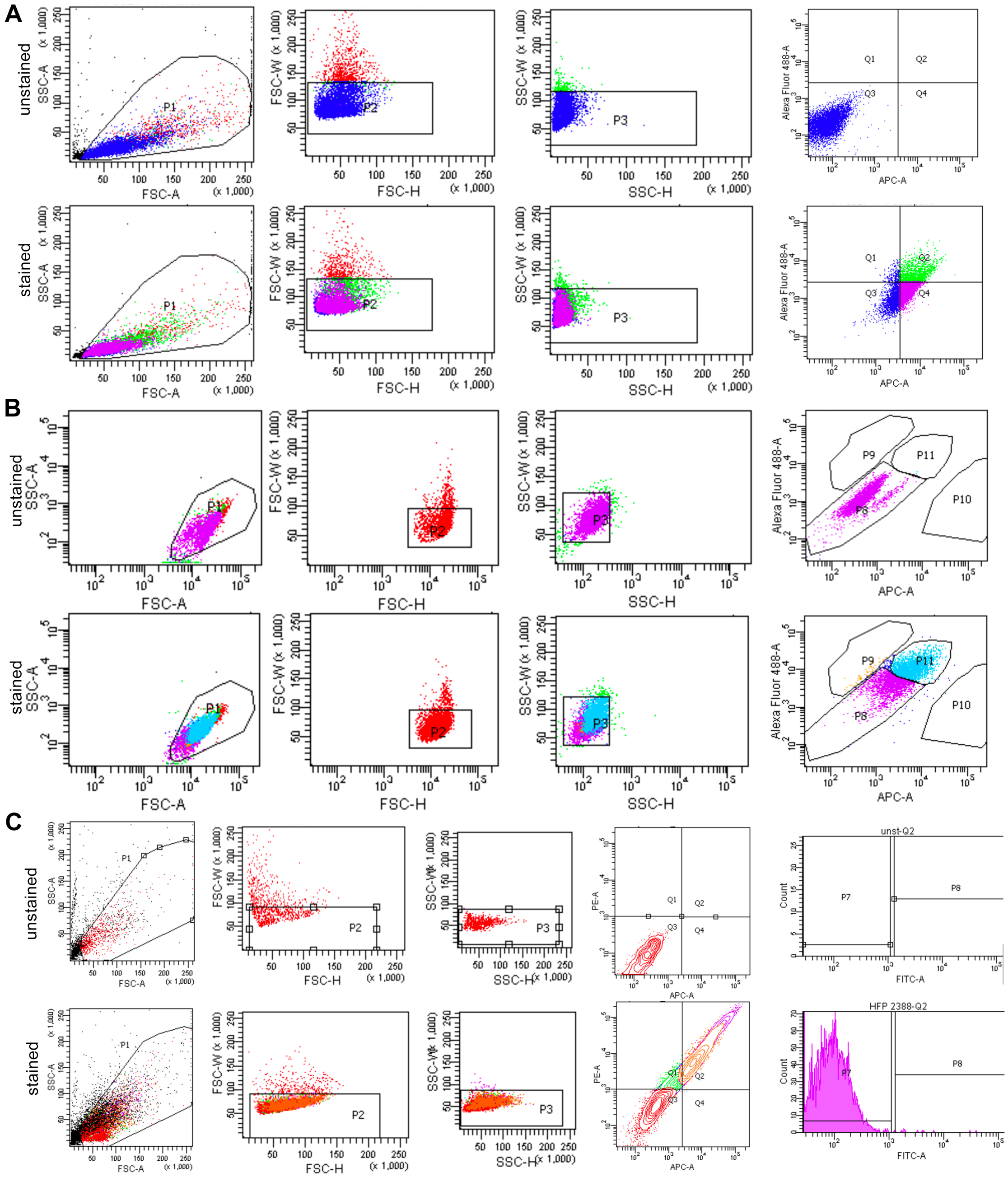

**Figure S1: Gating strategy**

Cells were first gated based on forward scatter (FSC-A) and side scatter (SSC-A) to exclude dead cells and potential cellular debris from the analysis (P1). Further gating was performed to remove duplets based on FSC-H/FSC-W (P2) and SSC-H/SSC-W (P3). Quadrant gating or polygonal gating was then drawn to exclude all events occurring in unstained cells for each channel (Alexa Fluor-488, APC, PE).

The same gating strategy was used to analyze: (**A**) the SOX9+/PTF1A+ cells; (**B**) the detection of SOX9+ cells by antibody or RNA probe (logarithmic scale); (**C**) the sorting of live SOX9+/PTF1A+ (Q2), either FGFR2- (P7) or FGFR2- (P8).

Gating was performed following the same criteria but independently for each sample to reflect differences between the analyzed populations.

## Supplementary Figure 2

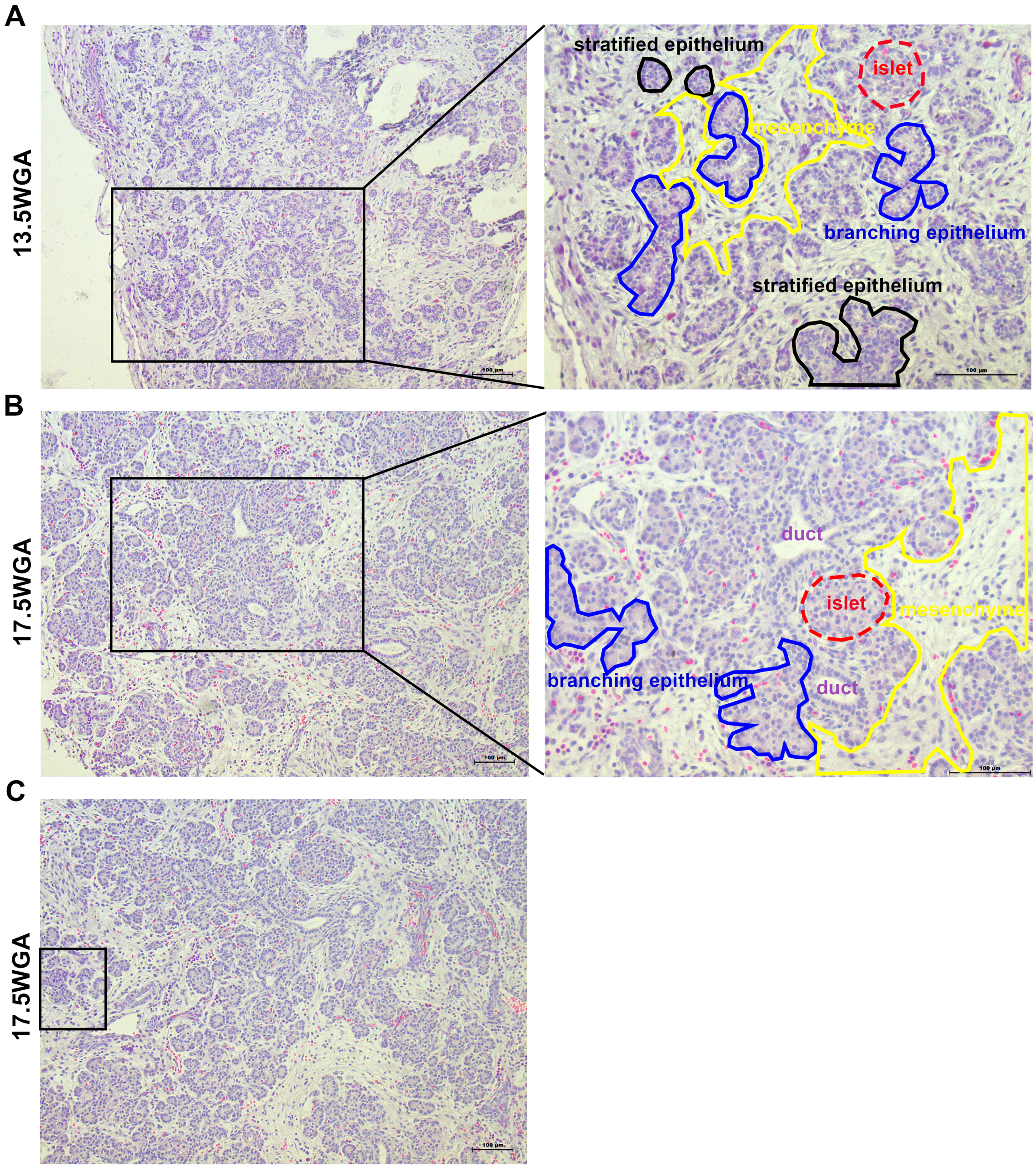

**Figure S2. Histological characterization of hFP at 13.5WGA and 17.5WGA.** (**A**) H&E staining of a representative section of hFP tissue at 13.5WGA, in which the pancreatic epithelium is intertwined with the surrounding mesenchyme. Right picture shows a higher magnification image of the panel indicated on the left. Different structures are identified including: stratified epithelium (black line), branching epithelium (blue line), pancreatic islet (red dashed line), mesenchyme (yellow line). (**B**) H&E staining of a representative section of hFP tissue at 17.5WGA. At higher magnification (right panel image), the following structures can be distinguished: branching epithelium (blue line), pancreatic islet (red dashed line), mesenchyme (yellow line), ducts. (**C**) Low magnification image of hFP tissue at 17.5WGA showing the presence of stratified clustered epithelium near the edge of the tissue (represented within the black square). Scale bar: 100 μm.

## Supplementary Figure 3

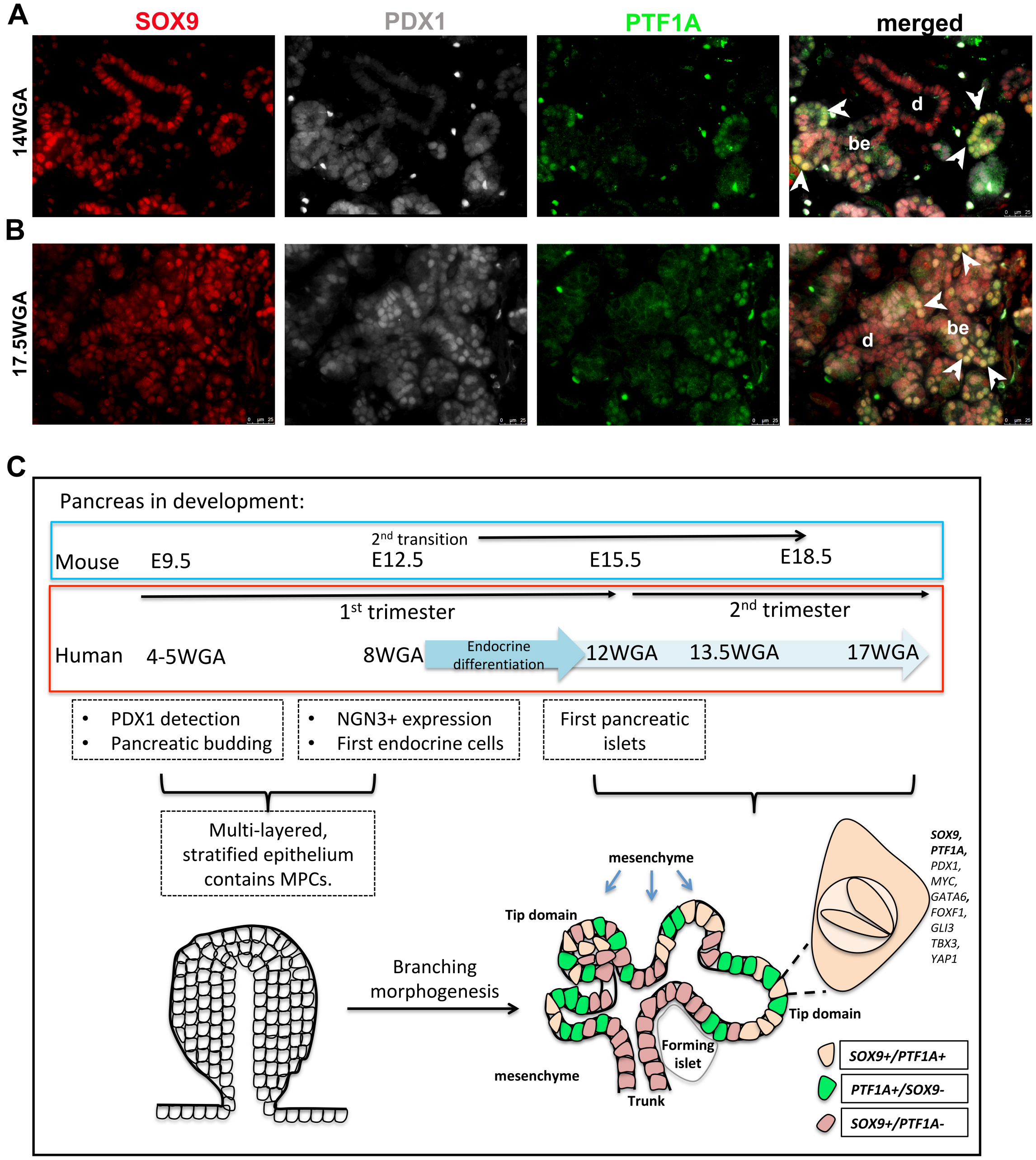

**Figure S3. Identification of tip cells co-expressing PDX1, SOX9, and PTF1A in the hFP of the second trimester.**

(**A-B**) Cells co-expressing SOX9 (red), PDX1 (grey) and PTF1A (green) at 14WGA (**A**) and 17.5WGA (**B**) can be identified at the tips of the branching epithelium (be) and can be distinguished from developing ducts (d), marked mainly by SOX9 expression and reduced PDX1 expression. Arrowheads indicate cells co-expression of the three markers. Scale bar: 25 μm. (**C**) Schematic illustration of the developmental timeline in human and mouse pancreatic organogenesis. Embryonic stages in mouse are compared to weeks of gestation in human. Main events are reported until second trimester in human development. Graphic visualization highlights the location where SOX9+/PTF1A+ cells are present within the branched epithelium in the second trimester after branching morphogenesis has started.

**Supplementary Figure 4**

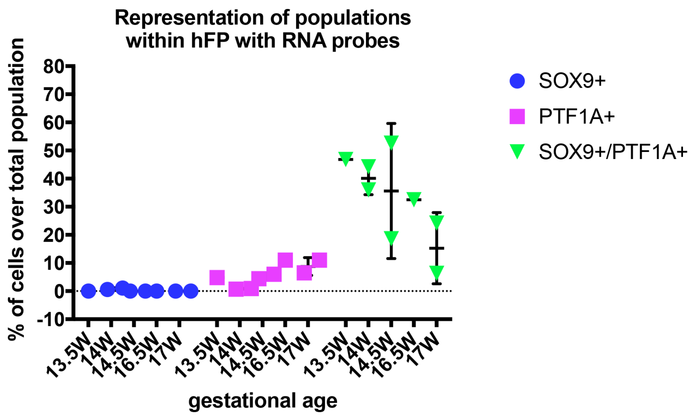


**Figure S4. Identification of cells co-expressing SOX9 and PTF1A in the hFP identified by RNA probes.**

Representation of SOX9+, PTF1A+, SOX9+/PTF1A+ cell subpopulations (individual plot of % of cells over total pancreatic digestion) isolated by RNA probes at different GA shows a decrease of the SOX9+/PTF1A+ pool over time, and a concomitant increase of PTF1A+ only cells. SOX9+ cells represent a minor fraction of the total population and display minimal change among the GA here analyzed.

## Supplementary Figure 5

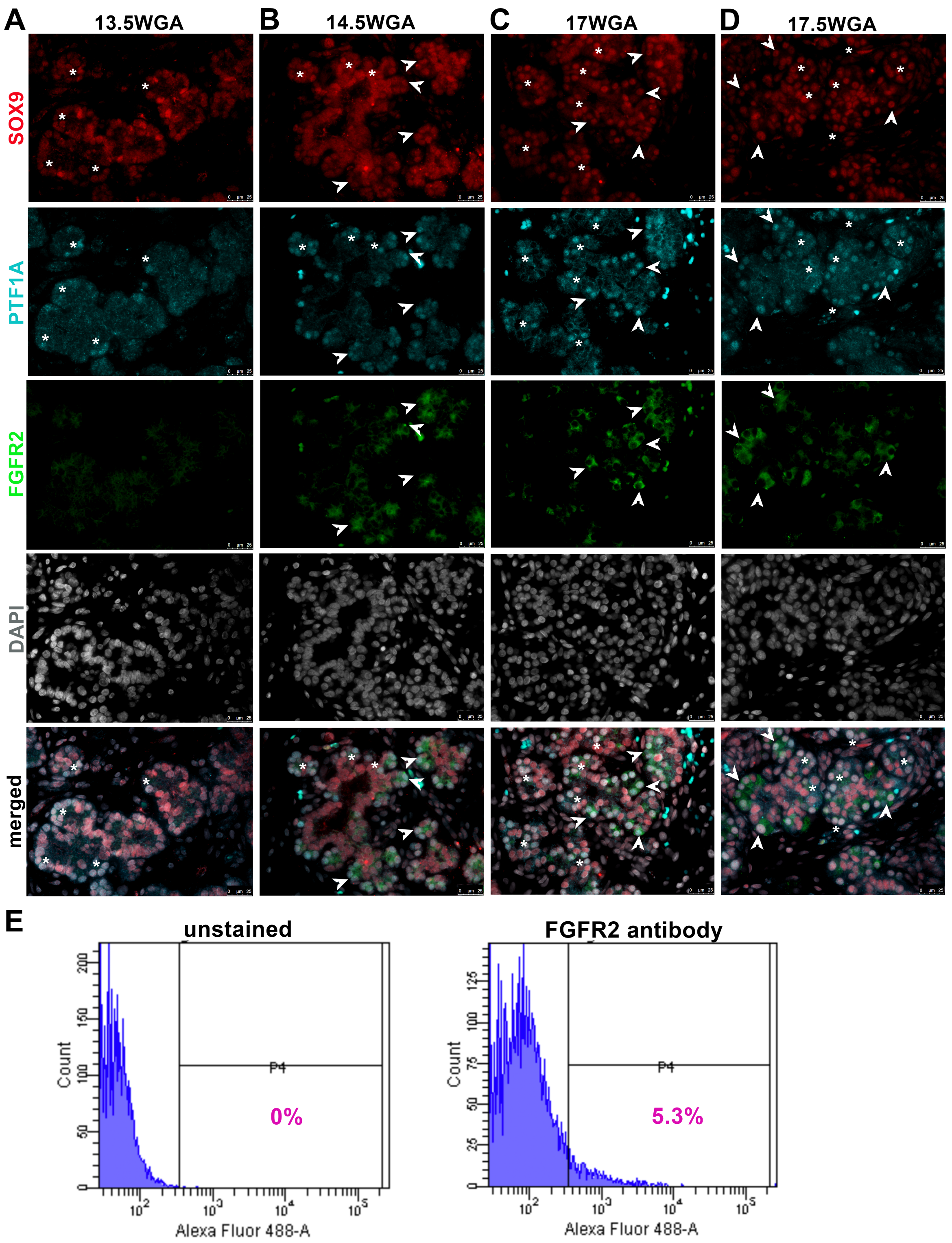

**Figure S5. Identification of cells co-expressing SOX9, PTF1A and FGFR2 in the hFP.**

(**A-D**) Cells co-expressing SOX9 (red), PTF1A (blue), FGFR2 (green) at 13.5WGA (**A**) 14WGA (**B**), 17WGA (**C**) and 17.5WGA (**D**) can be identified. Arrowheads in the merged images indicate cells co-expression of the three markers. Asterisks in the merged images indicate cells co-expressing SOX9 and PTF1A but not FGFR2. FGFR2 expression was almost undetectable at 13.5WGA but could be detected a later stages. Nuclei are represented in DAPI (grey). Scale bar: 25 µm. (**E**) The FGFR2 antibody used for FACS was further tested on primary total cells obtained from human fetal lung at 18.5WGA. Gating was established based on unstained control. 5.3% of cells resulted positive by FGFR2 staining.

# Supplementary Figure 6

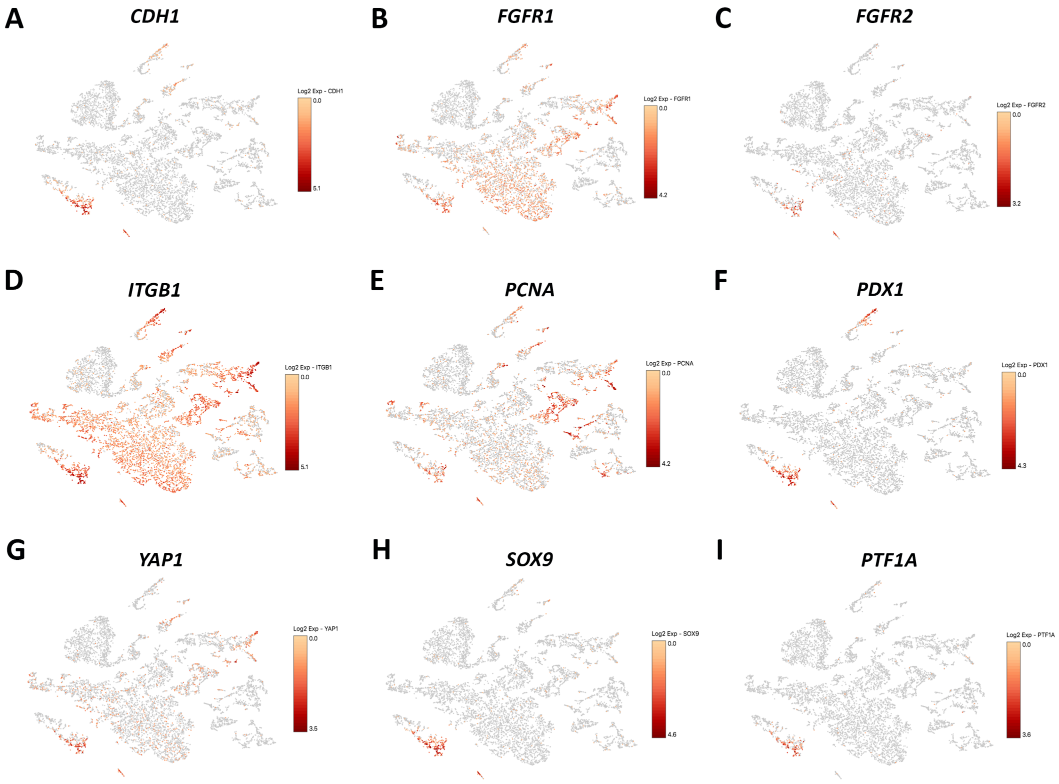


**J**

| Gene | Number positive | Percent Positive |
|---|---|---|
| CDH1 | 589 | 6.3 |
| FGFR1 | 2199 | 23.5 |
| FGFR2 | 188 | 2.0 |
| ITGB1 | 5393 | 57.6 |
| PCNA | 1995 | 21.3 |
| PDX1 | 498 | 5.3 |
| PTF1A | 200 | 2.1 |
| SOX9 | 454 | 4.8 |
| YAP1 | 916 | 9.8 |

**Figure S6: Expression of progenitor markers in a 15.4WGA hFP at the single cell level**

Expression of progenitor-specific markers in a hFP at 15.4WGA by scRNA-seq. tSNE plots are shown for: *CHD1* (**A**), *FGFR1* (**B**), *FGFR2* (**C**), *ITGB1* (**D**), *PCNA* (**E**), *PDX1* (**F**), *YAP1* (**G**), *SOX9* (**H**), *PTF1A* (**I**). **J**) Number and percentage of positive cells, over the total n=9,324 cells analyzed, for each listed marker.

## Supplementary Figure 7

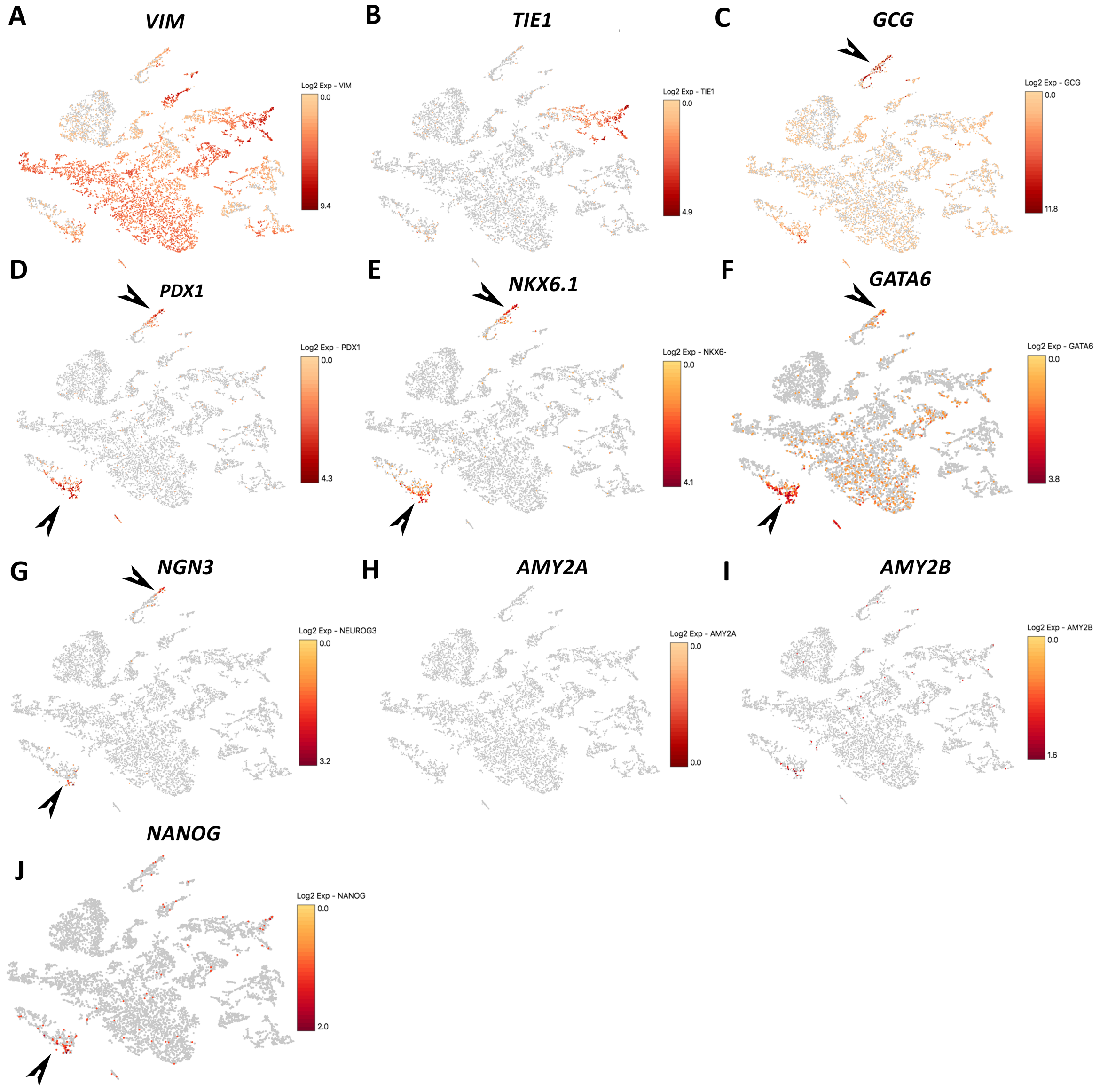

**Figure S7: Characterization of the cell clusters in the human developing pancreas by scRNA-seq**

Expression of lineage specific markers in a hFP at 15.4WGA by scRNA-seq is shown in tSNE plots for: *VIMENTIN* (**A**), *TIE2* (**B**), *GLUCAGON* (**C**), *PDX1* (**D**), *NKX6.*1 (**E**), *GATA6* (**F**), *NGN3* (**G**), *AMY2A* (**H**), *AMY2B* (**I**), *NANOG* (**J**).

**Supplementary Figure 8**

| Function | Genes |
|---|---|
| **Cell Morphology** | *ATF3, B2M, DLK1, GCG, IGF2, KRT18, KRT8, MT1F, NR4A1, RBPJ, REG1A, SERPINA1* |
| **Digestive System Development and Function** | *ATF3, ATP1B1, B2M, CEL, CLU, DLK1, EPCAM, GCG, HBA1/HBA2, IGF2, KRT18, KRT8, MDK, MT1F, NR4A1, REG1A, REG3G, SPINK1* |
| **Organ Morphology** | *ANXA4, ATF3, ATP1B1, B2M, CEL, CLPS, COL1A2, DLK1, GCG, HBA1/HBA2, HBB, IGF2, KRT18, KRT8, MDK, NR4A1, RBPJ, REG1A, SPINK1, SYCN* |
| **Organismal Development** | *AMBP, ATF3, B2M, CEL, CLU, COL1A2, DLK1, GCG, IGF2, KLK1, KRT18, KRT8, MCL1, MDK, MT1F, NR4A1, RBPJ, REG1A, SPINK1* |
| **Cell Death and Survival** | *ANXA4, ATF3, B2M, BTG2, CEL, CLU, CPA1, CTRB2, CY5BA, DLK1, DPEP1, EPCAM, GCG, GMNN, GP2, HBA1/HBA2, HBB, IGF2, KLK1, KRT18, KRT8, MALAT1, MCL1, MDK, MT1F, MT1X, MT2A, NR4A1, PRSS1, RBPJ, REG1A, SERPINA1, SPINT2, TMSB10/TMSB4X* |
| **Cellular Assembly and Organization** | *ATF3, CLU, GP2, KRT18, KRT8, MCL1, MT2A, SERPINA1, TMSB10/TMSB4X* |
| **Cellular Development** | *ATF3, B2M, BTG2, CEL, CLU, COL1A2, DLK1, DPEP1, EPCAM, GCG, GMNN, HBA1/HBA2, IGF2, KLK1, KRT18, KRT8, MALAT1, MCL1, MDK, MEG3, MT2A, NR3A1, NR4A1, RARRES2, RBPJ, REG1A, TMSB10/TMSB4X* |
| **Cellular Growth and Proliferation** | *ATF3, B2M, BTG2, CEL, CLU, COL1A2, DLK1, DPEP1, EPCAM, GCG, GMNN, HBA1/HBA2, IGF2, KLK1, KRT18, KRT8, MALAT1, MCL1, MDK, MEG3, MT1X, MT2A, NR4A1, RBPJ, REG1A, REG3G, SERPINA1, SPINT2, TMSB10/TMSB4X* |
| **Embryonic Development** | *ATF3, CLU, COL1A2, EPCAM, GMNN, IGF2, KRT18, KRT8, MCL1, MDK, MT1F, NR4A1, RBPJ* |
| **Organ Development** | *ANXA4, ATF3, ATP1B1, CLU, EPCAM, GCG, HBA1/HBA2, IGF2, KRT18, KRT8, MT1F, NR4A1, REG3G* |

**Figure S8: Gene list for the top 10 functions generated by IPA analysis of the *SOX9+/PTF1A+* cell cluster from scRNA-seq.**

IPA analysis of the significantly differentially expressed genes (p,0.05, FDR<0.05 and logFC<-1.5 or >1.5) between the SOX9+/PTF1A+ cluster and remaining pancreatic clusters. Main significant functions to pancreas development are here listed with respective genes for each specific function.
